# A Virtual Clinical Reasoning Case for Medical Students Using an Ophthalmology Model: A Case of Red Eye

**DOI:** 10.15766/mep_2374-8265.11117

**Published:** 2021-03-04

**Authors:** Nisha Chadha, Douglas Fredrick, Alefiyah Malbari, Joanne Hojsak

**Affiliations:** 1 Assistant Professor of Ophthalmology and Medical Education, Icahn School of Medicine at Mount Sinai/New York Eye and Ear, Eye and Vision Research Institute; 2 Deputy Chair of Education and Professor of Ophthalmology, Icahn School of Medicine at Mount Sinai/New York Eye and Ear, Eye and Vision Research Institute; 3 Assistant Professor of Pediatrics and Medical Education, Icahn School of Medicine at Mount Sinai; 4 Professor of Pediatric Critical Care Medicine and Medical Education, Icahn School of Medicine at Mount Sinai

**Keywords:** Ophthalmology, Red Eye, Preclinical Medical Education, Clinical Reasoning, Virtual Learning, Case-Based Learning

## Abstract

**Introduction:**

Triggered by the COVID-19 pandemic, medical education has moved online, tasking medical educators with developing virtual learning experiences. This is particularly challenging for less-represented disciplines, such as ophthalmology. We designed a red eye clinical reasoning case for preclinical medical students, which can be delivered virtually, using video conference software.

**Methods:**

We developed a 90-minute red eye/clinical reasoning workshop for which prereading was assigned to students. We then delivered a virtual development session to nonophthalmologist copreceptors and provided a session faculty guide. The entire first-year medical student class (No. = 140) participated in one of four identical workshops, which included virtual small- and large-group discussions. Students completed a knowledge pre- and posttest, and an optional session postsurvey.

**Results:**

Knowledge gains from pretest (No. = 94) to posttest (No. = 73) were statistically significant (*p* < .05), with average scores improving from 57% to 70%. Overall, students were satisfied, rating the following items 4 or 5 out of 5: session (86%, No. = 31), virtual format (83%, No. = 30), and if they recommended future use (69%, No. = 35).

**Discussion:**

This novel, virtual clinical reasoning case simulated small- and large-group learning, achieved knowledge gains, and was well received by students. Minor technical challenges were encountered but successfully remedied, without apparent disruption to learning. This virtual medical education model can be used to enhance ophthalmology education in preclinical medical students and can be adapted for virtual design of other curricular content.

## Educational Objectives

By the end of this activity, learners will be able to:

1. Demonstrate clinical reasoning skills in the context of an ophthalmologic chief complaint of eyelid swelling.
2. Create a history of present illness characterization complete with representative external images for the common causes of red eye.
3. Develop a differential diagnosis for red eye complaints.
4. Apply team-learning strategies using a remote learning platform.

## Introduction

A 2014 survey of US medical schools found that while direct ophthalmoscopy was taught at most schools, additional preclinical ophthalmology education was variable, and required clinical ophthalmology experiences had significantly declined.^1^ Consequently, a survey of medical students at one institution found that 50% were uncomfortable checking visual acuity, and 64% were uncomfortable diagnosing eye emergencies.^2^ As the growing population ages, the need for eye health care will only increase, and it is important that graduating medical students gain skills in assessing ocular complaints. To provide guidance on content areas, the Association of University Professors of Ophthalmology proposed ophthalmology-related learning objectives for all graduating medical students, which include developing skills related to evaluation of common ophthalmic presentations such as red eye and vision loss.^3^ Familiarity with these presentations is important as ophthalmic chief complaints are common in the primary care setting and can comprise 5%-19% of visits.^4^

Encouragingly, ophthalmology modules, particularly in a problem- or case-based format, have been shown to increase medical student comfort, satisfaction, and knowledge. Hoonpongsimanont and colleagues reported that a 40-minute ophthalmic exam teaching session improved student comfort with the exam.^5^ In another study, Turkish medical students preferred a team-based learning cornea module to didactic lectures.^6^ In China, students performed better on a problem-based learning eye module when compared to those receiving a didactic module.^7^ These studies suggested that brief teaching sessions can improve student ophthalmic knowledge and skills. While *MedEdPORTAL* offers educational materials related to the slit lamp and fundus examination,^8–11^ at present it does not include resources on approaches to red eye examination.

The urgent and abrupt transition of medical education to online learning in the setting of the COVID-19 pandemic, has tasked medical educators with rapidly developing virtual learning experiences. This circumstance has created an additional challenge for disciplines such as ophthalmology, within which there may be fewer medical educators, and for which content is less represented in the curriculum. To address this content need, and need for virtual educational tools, we designed a red eye clinical reasoning case for preclinical medical students, which can be delivered virtually, using an online meeting platform, such as Zoom. This module served a dual purpose of developing clinical reasoning skills while increasing ophthalmic education, delivered in an interactive flipped classroom format.

## Methods

We developed a 90-minute red eye clinical reasoning workshop as part of the clinical skills course, the Arts and Science of Medicine, at the Icahn School of Medicine at Mount Sinai. This session was conducted towards the end of the first-year medical student (MS 1) academic year, at which point students had learned history taking and the head and neck physical exam, and therefore was a useful exercise in clinical reasoning, differential diagnosis generation, and determination of focused physical exam. In an effort to increase experience with interactive, virtual learning, and early exposure to ophthalmology, MS 1s, who had recently transitioned to online learning in the setting of the COVID-19 pandemic, were the targeted cohort.

Faculty from the Icahn School of Medicine at Mount Sinai's Department of Ophthalmology also involved in medical education served as content experts. We assigned prereading articles on red eye and swollen eyelid to the students 1 week prior to the session.^12,13^ We provided faculty copreceptors in our clinical skills course with a faculty guide (Appendix A) describing the session, followed by a 1-hour virtual development session. The MS 1 class was divideokd into four groups, and participated in one of four identical workshops, which were led by the content experts and cofacilitated by three to five faculty preceptors. Prior to the session, Google Docs for each session were created and shared with students assigned to each session. The four online meetings (Zoom) were prepopulated with breakout room participants and polls.

At the beginning of the session, students completed a five-question pretest on red eye, using Google Forms (Appendix B). Pretest questions were in the form of red eye clinical vignettes, accompanied by an image, with multiple-choice responses and assessed content which was covered in the prereading. The questions were written and agreed upon on by content experts. After providing students with an overview of the session and learning objectives, students were asked to generate a differential diagnosis for the red eye. A copreceptor transcribed the participants' list using the virtual whiteboard function on Zoom, which allowed all participants to follow along.

Next, students worked in preassigned small groups of two to four and used online resources (such as search engines) to develop history of present illness descriptions for common causes of red eye and identify a representative image online. They were instructed to paste their narrative and image into a shared Google Doc (Appendix C). The breakout room function on Zoom was used to virtually divide students into small groups for this activity, after which they returned to the large group. Faculty preceptors were assigned to individual breakout rooms to assist with this activity. Back in the large group, the content expert projected the completed Google Doc using the share function in Zoom, and reviewed each description, providing feedback and answering questions.

Following this exercise, content experts led the students as a large group through the clinical reasoning case, a case of eyelid swelling, using a PowerPoint presentation (Appendix D) which was shared on the meeting screen. We asked students to generate a differential and ask questions to refine the differential. Their responses to these prompts were elicited verbally or nonverbally using the chat function. After providing additional history, we prompted them to discuss what focused physical exam would be relevant for this chief complaint. We then shared the physical exam in the PowerPoint presentation, which included an external image of the eyes. A Zoom poll inquiring about the most likely diagnosis was administered, and results were shared and discussed. At this point the working diagnosis of preseptal cellulitis was reviewed, along with management and follow-up plans. The case progressed, and students were alerted that the patient called with worsening symptoms and was asked to come in for an urgent follow-up visit. We then shared the follow-up physical exam revealing progressive eyelid swelling, worse vision, motility disturbance, and proptosis concerning for orbital cellulitis. Case conclusions were discussed. Students then completed a posttest and optional postsurvey, and test questions were reviewed (Appendices B and E).

### Assessment

Student knowledge gains were assessed through administration of a pretest and posttest, which students were encouraged to complete but did not count towards the course grade. The pre- and posttests were identical, and each item was constructed in the form of a clinical vignette and representative image, followed by a query of the most likely diagnosis. An optional postsurvey containing three questions, rated on a 5-point Likert scale, was launched as a poll through Zoom at the end of the session.

Following the session, pretest, posttest, and postsurvey data were analyzed using Student's *t* test. The study was determined to be exempt by the Icahn School of Medicine at Mount Sinai Institutional Review Board.

## Results

While this was a required session for all MS 1 students (No. = 140), approximately 5% did not participate due to connectivity issues or excused absences. Of participating students, 94 completed the pretest, and 73 completed the posttest. There was variability in response rates on the pre and posttests due to difficulty accessing the Google form link to the test, or in some sessions, time constraints. Despite these barriers, unpaired *t* tests revealed that knowledge gains from pretest (No. = 94) to posttest (No. = 73) were statistically significant, with average scores on the five-question test improving from 57% to 70% (*p* < .05).

Subanalyses of each pre-/posttest item revealed that students' correct identification of anterior uveitis, blepharitis, and acute angle closure improved after the session from 20% to 60%, 56% to 77%, and 70% to 93%, respectively. However, surprisingly, correct recognition of subconjunctival hemorrhage and herpetic keratitis slightly decreased following the session, from 73% to 64% and 67% to 53%, respectively (Table 1). The most common incorrect response for the subconjunctival hemorrhage question was episcleritis, and for the herpetic keratitis question was corneal abrasion.

**Table 1.**
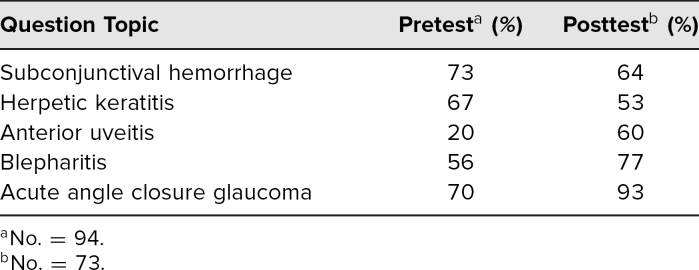
Percent of Correct Responses to Knowledge Questions

Survey responses to the optional postsurvey were limited as the poll could not be displayed during two of the workshops, and data from one session could not be recovered. In the one remaining session there were 39 students, of which 36 students responded (Table 2). On the 5-point scales, the majority of students provided a level 4 or 5 rating for the session (86%, No. = 31), the virtual format (83%, No. = 30), and the recommendation of future use (69%, No. = 25).

**Table 2.**
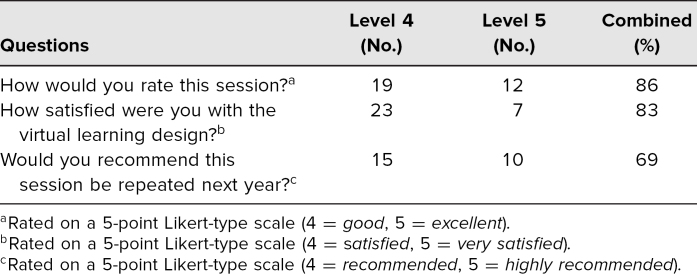
Postsession Evaluation Survey Responses (No. = 36)

## Discussion

This novel, virtual clinical reasoning case simulated small- and large-group learning and was well received by students. The 90-minute session was effective in increasing student ophthalmic knowledge, specifically in regard to recognition of common presentations of red eye. The session was conducted towards the end of the MS 1 academic year shortly following instruction on the head and neck exam and at which point students had gained experience with history taking. Because the workshop emphasized skills in clinical reasoning, differential diagnosis generation, and determination of focused physical exam, it may also be useful for second-year medical students.

With regard to knowledge gains, while there was an overall improvement in scores from pre- to posttests, interestingly students incorrectly changed their answer to the questions related to subconjunctival hemorrhage and herpetic keratitis from pre- to posttest. This change in response for these two items may be due to variability in emphasis placed on these entities between workshops, as they were led by two different faculty members. The finding also suggested that more specific characterization and distinction of these entities from one another is needed in the future.

Overall student response to the session was positive, but only 69% recommended its future use. While narrative comments were not collected as part of the survey, we believe that students may have been less likely to recommend future use due to discomfort with the virtual learning format and features, as this was one of their first interactive virtual learning sessions during the peak of the pandemic in New York City.

### Limitations

While this workshop was successful, there were challenges faced and lessons learned that can be applied to improve future implementation of the sessions. Technical challenges including breakout room and poll failure, and connectivity issues were encountered during some of the sessions. Consequently, not all students were able to participate in the breakout rooms, or complete the postsurveys, leading to the reduced response rate on the postsurvey. However, encouragingly, these challenges were navigated successfully in real time with minimal disruption to the session. To circumvent faculty connectivity or audio issues, all faculty preceptors were made cohosts on the meeting so that they could take over meeting functions. Additionally, while the breakout room function did not work for two of the sessions, students were able still to work in small groups within the meeting using the private chat function or edit functions in the Google Doc. Additionally, a small group of students had difficulty joining the meeting, but sessions were recorded using the record function, so they could be shared afterwards. As with live, in-class teaching, pitfalls should be anticipated, and contingency plans considered. Finally, despite a faculty development session reviewing the Zoom features, there was variability in faculty comfort using the platform. We anticipate improvement in web conference proficiency and decreased technical challenges as both students and faculty become more experienced with the web conferencing platform and virtual learning. Faculty practice simulations of Zoom or other web conferencing features in advance of the session may also be considered.

Data collection were limited due to the online format of collection and time constraints. Specifically, postsurvey data were limited due to poll function failure and inability to recover some poll data from the recorded sessions. However, the postsurvey data presented were all from one workshop, with a relatively high response rate within the session. In the future, we recommend against using the poll function for research-related data collection in order to maximize data obtained. Pre- and posttest data collection were limited by time constraints, and can be improved with better time-keeping throughout the session.

Another factor worth noting was that these sessions were led by ophthalmologists with a special interest in medical education. While the sessions can certainly be led by nonophthalmologists, additional preparation prior to the session may be required. Alternatively, ophthalmologists with an interest in education could be recruited to facilitate this workshop. Ophthalmology departments that have a faculty member who serves as vice chair of education or the director of medical student education can potentially rely on these individuals to provide support for such a session.

Finally, virtual meeting creation including prepopulation with breakout rooms and polls on Zoom, along with Google Doc creation and sharing was somewhat time consuming. Therefore, administrative assistance with the preparation can be helpful.

### Conclusions and Future Directions

We presented a virtual medical education model specifically involving clinical reasoning related to red eye. The session can be modified, and we encourage educators to do so, based on the local learning needs and objectives. For example, the clinical reasoning portion could be presented as a standalone session, if time is a limitation. This model can be used to enhance preclinical ophthalmology education, and can also be adapted for virtual design of other problem or case-based curricular content using Zoom or other web-conferencing platforms.

## Appendices

Faculty Guide.docxPre- and Posttest.docxTemplate for Google Document.docxRed Eye Clinical Reasoning Presentation.pptxRed Eye Session Polls.docx
All appendices are peer reviewed as integral parts of the Original Publication.
