# Supplementary material for: A Virtual Clinical Reasoning Case for Medical Students Using an Ophthalmology Model: A Case of Red Eye
Source: MedEdPORTAL. 2021 Mar 4;17:11117. doi: 10.15766/mep_2374-8265.11117 (PMC7970637; doi:10.15766/mep_2374-8265.11117)
Supplement: Supplementary file 1 — Faculty Guide.docxPre- and Posttest.docxTemplate for Google Document.docxRed Eye Clinical Reasoning Presentation.pptxRed Eye Session Polls.docx [file mep_2374-8265.11117-s001.zip › A. Faculty Guide.docx]

FACULTY GUIDE

Virtual “Red Eye” Clinical Reasoning Workshop for Medical Students using ZOOM

Nisha Chadha, MD, Douglas Fredrick, MD Alefiyah Malbari, MD, Joanne Hojsak, MD

**VIRTUAL “RED EYE” CLINICAL REASONING Workshop for Medical Students using ZOOM**

**Session Overview for Faculty**

Total time: **90 minutes**

1. [Intro](#intro) and [pre-test](#Pretest) – (2+8 minutes)
2. [Red eye differential diagnosis and generation of History of the Present Illness (HPI)/external exam description](#part2) (35 minutes)
3. [Clinical reasoning case intro](#part3)/[Develop differential diagnosis](#ddx) and [questions to refine differential](#questions) (5 minutes)
4. [Remaining History key physical exam](#part4), [Exam photos](#examfindings), [diagnosis and management](#mgmt1) (15 minutes)
5. [Follow up visit history](#part5), [physical exam findings](#exam2), [differential diagnosis](#mgmt2) (10 mins)
6. [Case conclusion – work up and management](#part6) (5 minutes)
7. [Complete post-test and review answers](#part7) (10 minutes)

***Pre-reading***

1. Galor A, Jeng BH. Red Eye for the Internist: When to Treat, When to Refer. Cleve Clin J Med. 2008;75(2):137-44.
2. Carlisle RT and Digiovanni J. Differential Diagnosis of the Swollen Red Eyelid. Am Fam Physician. 2015;92(2):106-12.

**Pre-planning:**

1. Zoom pre planning:

- Group class into large groups of ~35-40 students
- Create session meetings in Zoom
- Designate faculty preceptors as co-hosts on Zoom meetings
- Pre-create polls in Zoom meetings
- Pre-create Google Docs (1 for each session)
- Pre-assign students within each session into smaller groups using “Breakout Room” setting in Zoom. Assign 1-2 “red eye” topics on Differential Diagnosis slide to each group, depending on number of groups.
- Create and provide links for pre-test and post-test

**INTRODUCTION (2 min)**

**Review objectives of session:**

- By the end of this session, students will be able to:
  - Develop clinical reasoning skills in the context of an ophthalmologic chief complaint of eyelid swelling.
  - Create an HPI characterization complete with representative external images for the common causes of red eye.
  - Practice medical history questions to refine the differential diagnosis for red eye complaints
  - Practice strategies for team learning using a remote learning platform.

**1.** **PRETEST (8 mins)**

- Please allow students 10 minutes to complete the 5-question pre-test *(pre-test can be created using Google Forms and link can be displayed in chat function, posted on learning platform, or emailed)*

**2. RED EYE DIFFERENTIAL DIAGNOSIS and Generation of BASIC HPI/External exam description (35 mins)**

- **As a large group,** generate a differential diagnosis for red eye (based on pre-reading). A faculty preceptor can **scribe differential using “Whiteboard” function on Zoom.**
  - *Next, show sample differential slide (below).* **Ask students** if there is anything they would **add or remove** from this list. They can type suggestions into chat box.

*
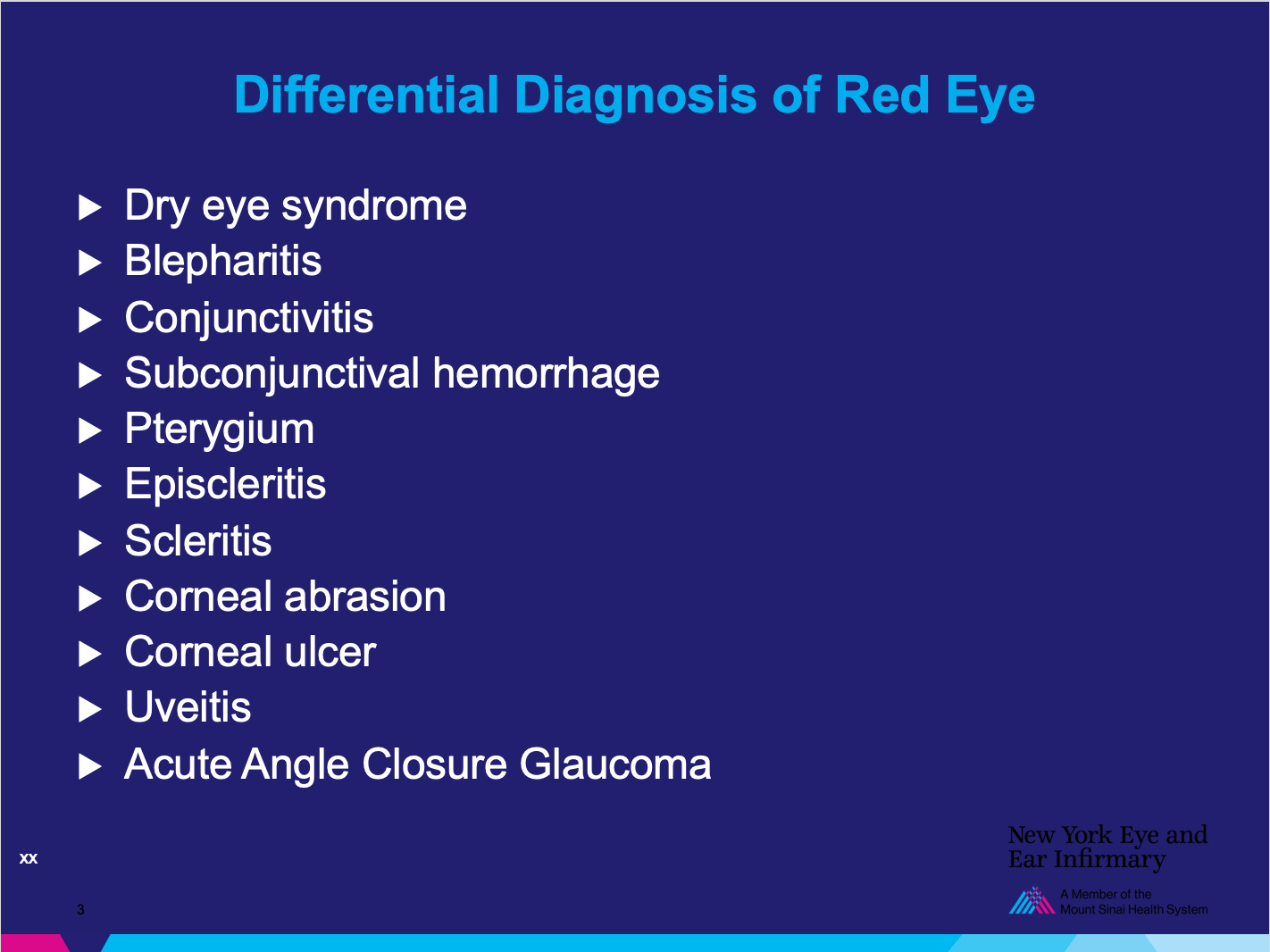
*

- Break into small groups by clicking “Breakout Room” button on Zoom meeting menu bar. Faculty preceptors can join individual groups. *Any students that aren’t automatically assigned to pre-designated rooms can manually be assigned to their room.*
- Each small group should assign one student to be the representative (student will relay info back to large group).
  - Each small group will generate an HPI and external exam description for 1-2 items on the differential and **type this into the large group Google Doc.** *The Google Doc will become a “study guide” for the students!* The description should cover the following:
    - Possible chief complaint

Presenting history and associated symptoms

- - - Physical exam:  students search online for a representative **image** of the diagnosis and paste it into Google Doc
  - **Lead Preceptor:** Close breakout rooms *(Zoom automatically generates 1 minute warning when you close group).* Everyone returns to large group on zoom.
  - The lead preceptor now **“shares” Google Doc using “share” button** on Zoom menu bar. Each student rep will read their “HPI description” and image. Lead preceptor provides feedback on descriptions.

**3: CLINICAL REASONING CASE and DEVELOPMENT of DIFFERENTIAL DIAGNOSIS and QUESTIONS to REFINE DIFFERENTIAL (5 mins)**

- Next, Lead preceptor reads the following stem to the students (and shows **slide A**):

**
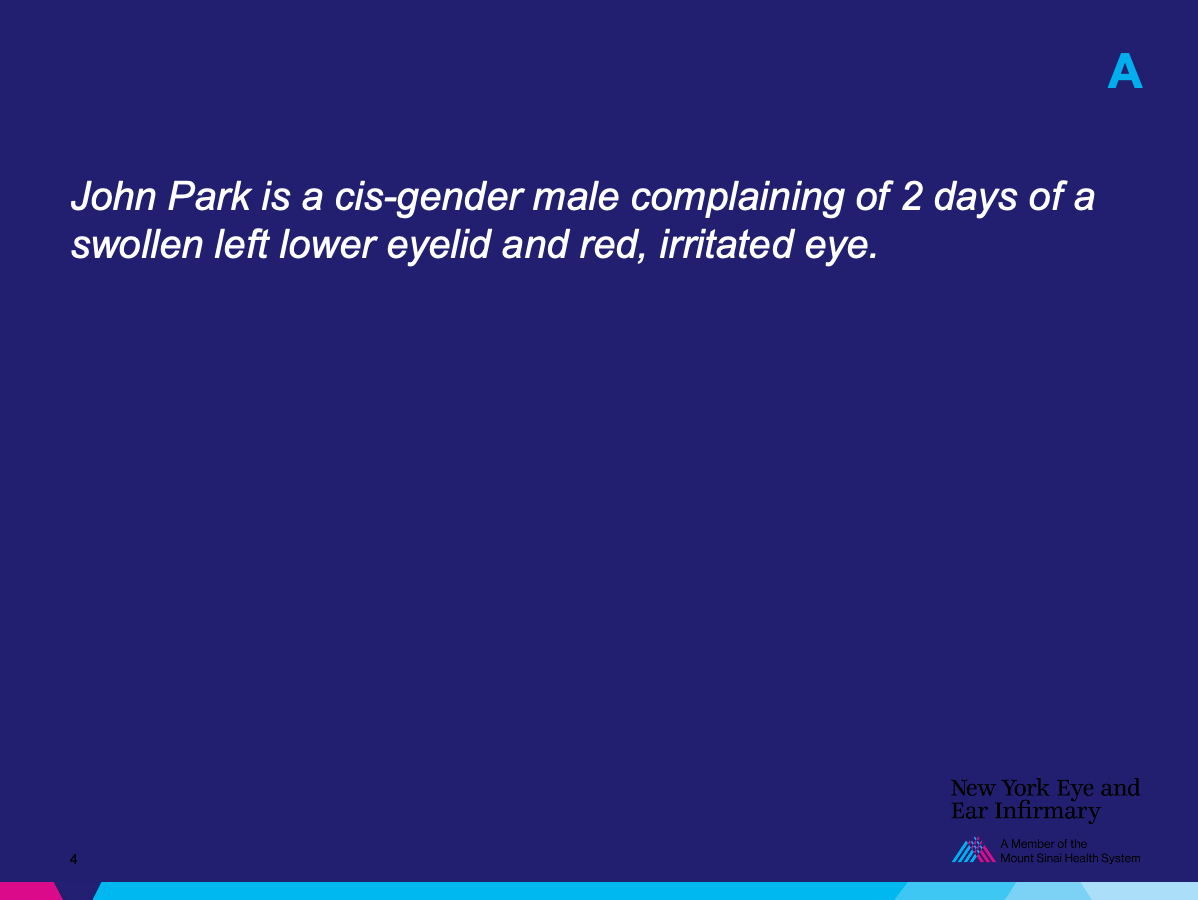
**

**A. Ask students to develop a differential diagnosis** and enter possible considerations into **Chat function** (can use online resources).

- - *Sample differential:*
    - Blepharitis
    - Chalazion
    - Preseptal cellulitis
    - Orbital cellulitis
    - Dacyrocystitis
- Co-faculty preceptor can again scribe using “Whiteboard” function*. (A new whiteboard can be created for this list)*
- **Lead preceptor** will summarize responses

**B. Generate questions to refine the differential:**

- Next ask students to **generate questions** to refine the differential and type them into **chat** function
- Co-faculty preceptor can also scribe these responses on “Whiteboard” and Lead preceptor can review
- *Possible questions:*
  - Is there a change in vision?
  - Is there discharge?
  - Is there eye or eyelid pain? Quality of pain – sharp or dull?
  - Contact lens wear?
  - Trauma?
  - Previous episodes?
  - History of ocular problems? Ocular surgery?
  - Associated symptoms? Fever? Sinusitis?

**4. REMAINING HISTORY (15 mins)**

- **Show slide B** (ask student to read aloud):


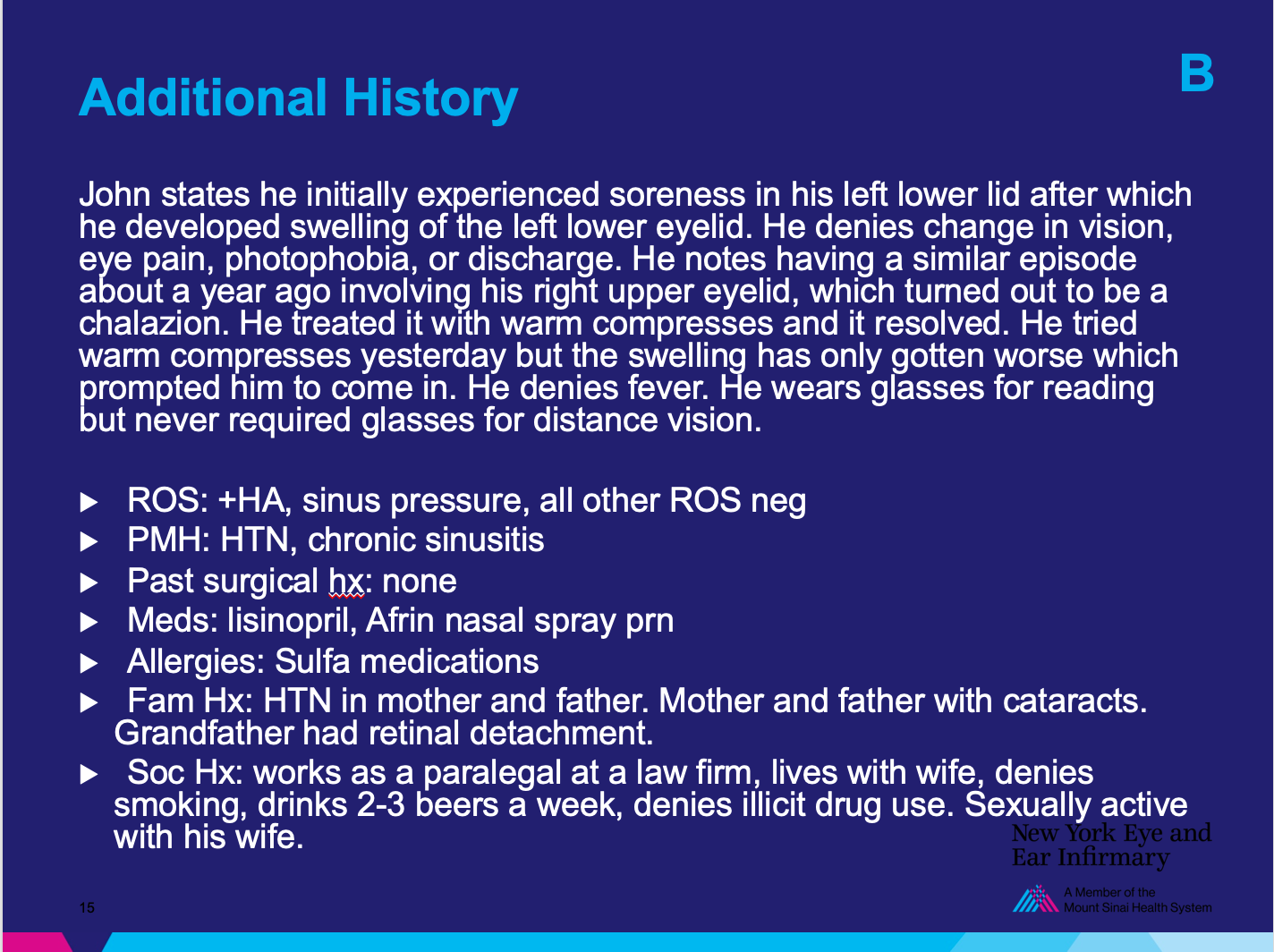


- **Lead preceptor asks, “What exam should we perform?”:** (have students type into chat, Lead preceptor verbally summarizes OR can scribe on whiteboard again)
  - *Possible responses: vitals, head, eyes, ears, nose and throat exam (HEENT) (vision, pupils, EOMs, penlight exam of eye, fluorescein staining of cornea)*
  - *check for preauricular lymph nodes, palpate sinuses, evaluate nasal mucosa*
- Share exam findings with students (Show **slide C**):


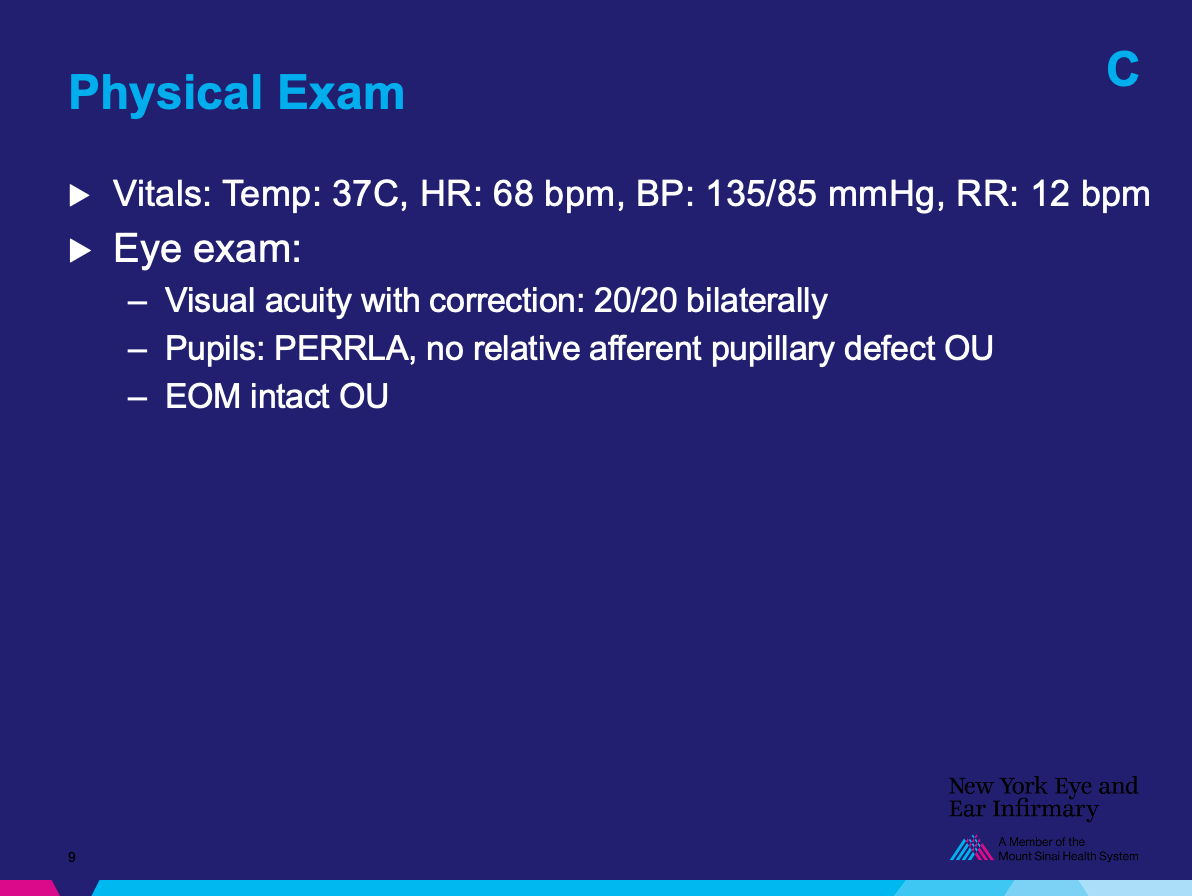


- - External exam: ***(show photo on Slide D)***


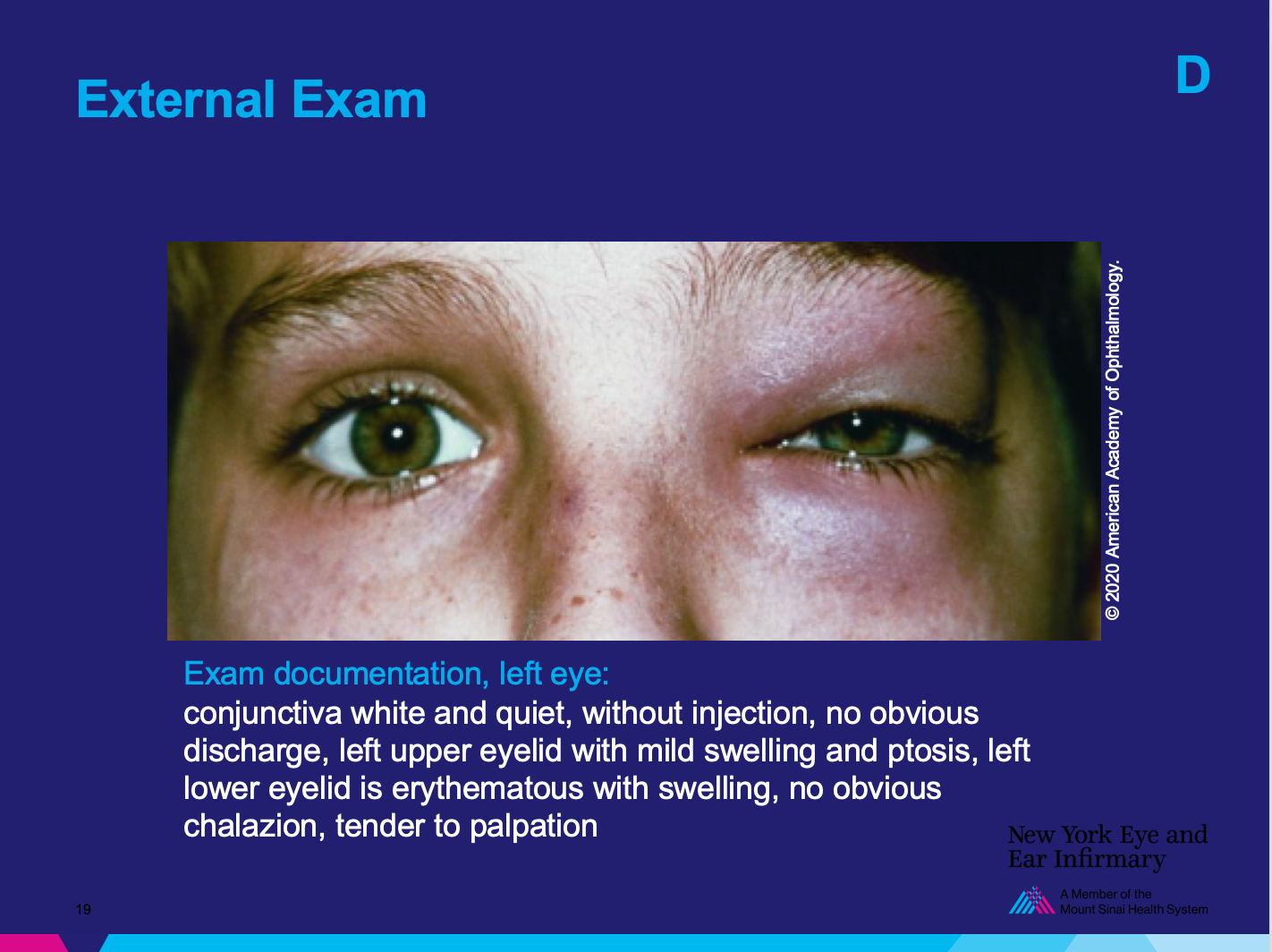


- - *Allow students 30 seconds to think about how they would describe the image, then ADVANCE to next slide which displays description below*
  - Share additional physical exam information **(Show slide E):**


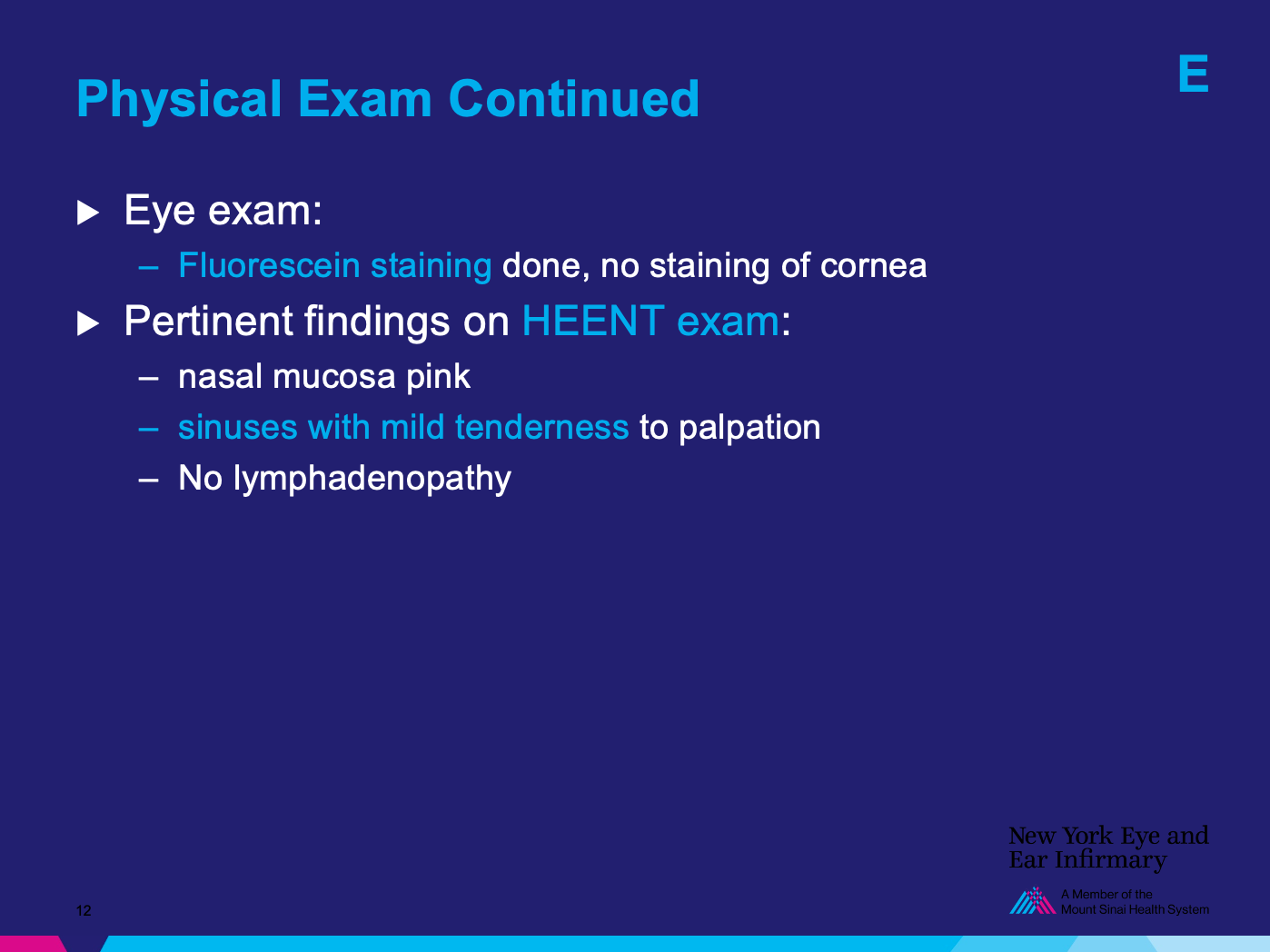


- **Lead preceptor displays Poll #1:** poll students on what they think is the most likely diagnosis is (Preseptal cellulitis). **Share results** on screen. Compare answers and discuss why presetpal cellulitis is most likely. *(Note: Poll question is “What is the most likely diagnosis?” Multiple choice response options are: blepharitis, chalazion, preseptal cellulitis, orbital cellulitis, and dacryocystitis)*
- Use chat function for students to ask questions
- Share treatment plan **(Display slide F):**


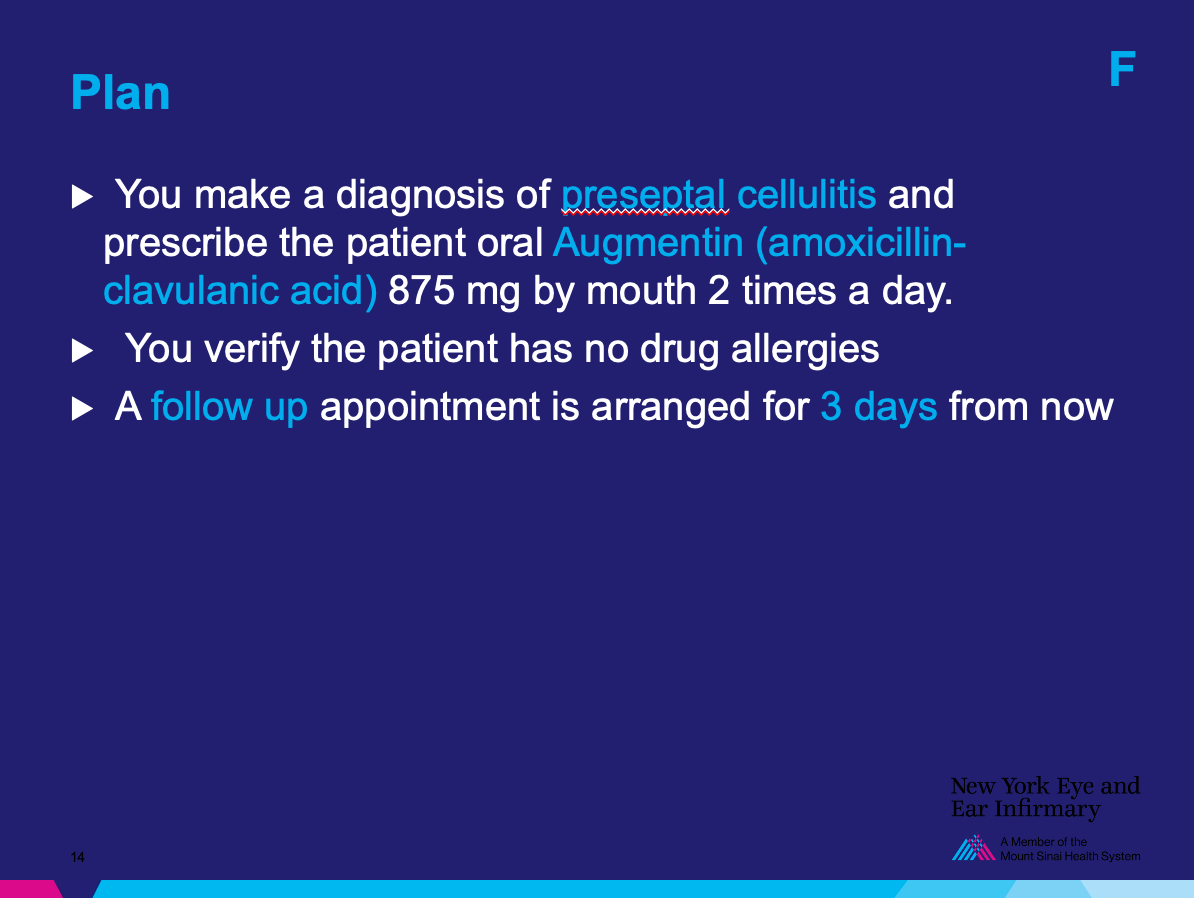


**5. FOLLOW UP VISIT HISTORY and FINDINGS (10 mins)**

**Follow up phone call (show slide G** and have someone read aloud):


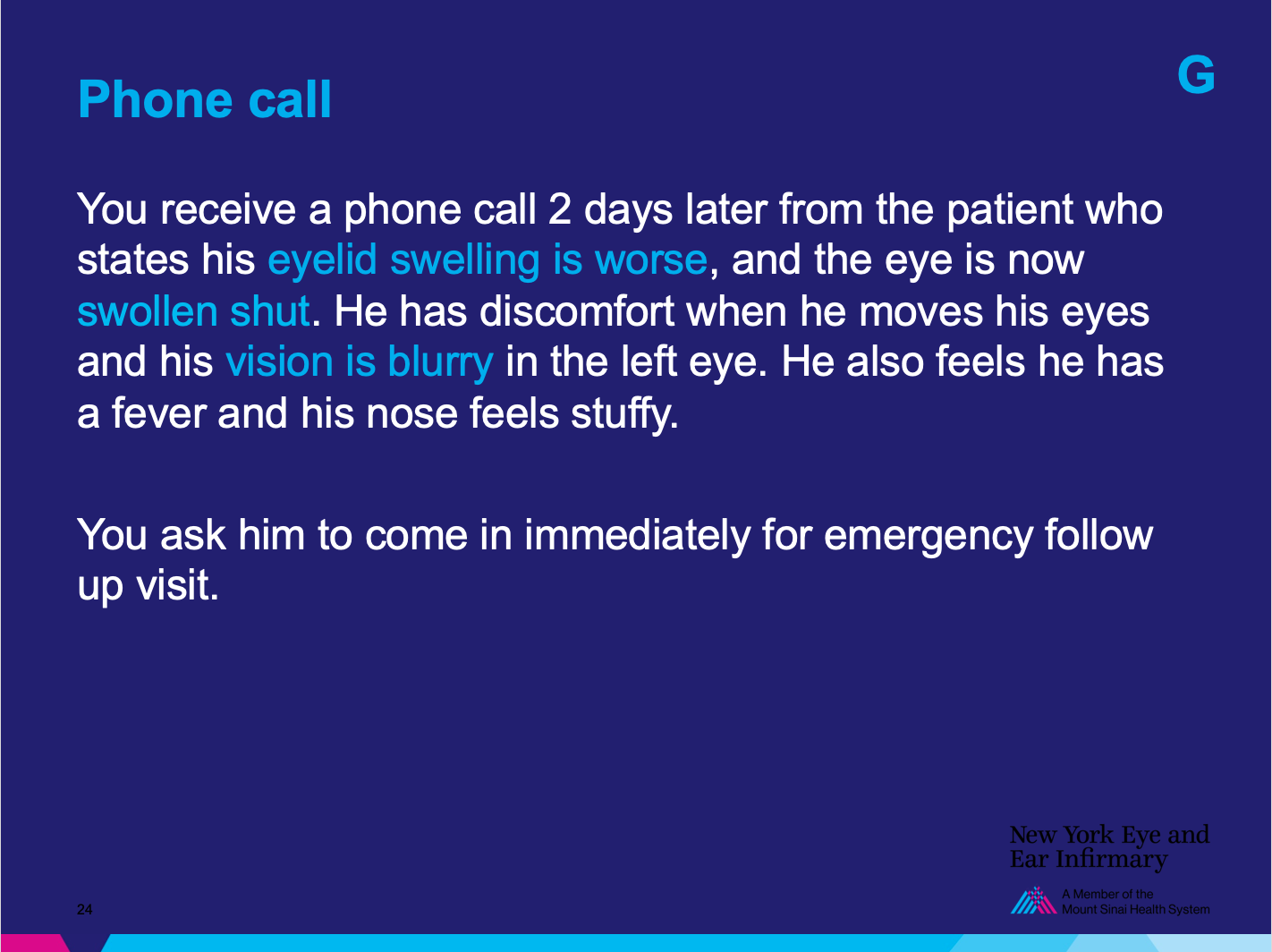


- Say, you perform a focused exam which reveals the following **(show slide H):**


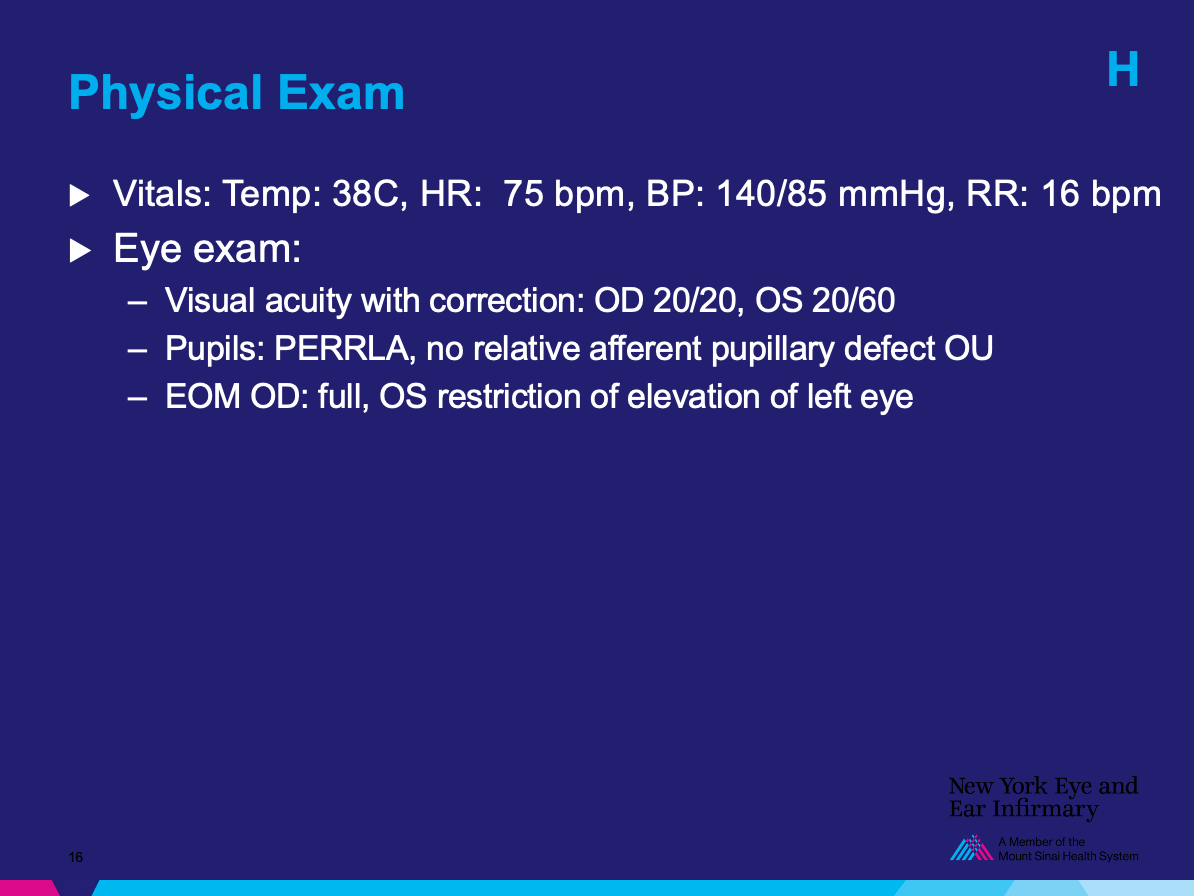


- External exam **(show slide I** *and* *allow students 30 seconds to consider how to describe/document*)


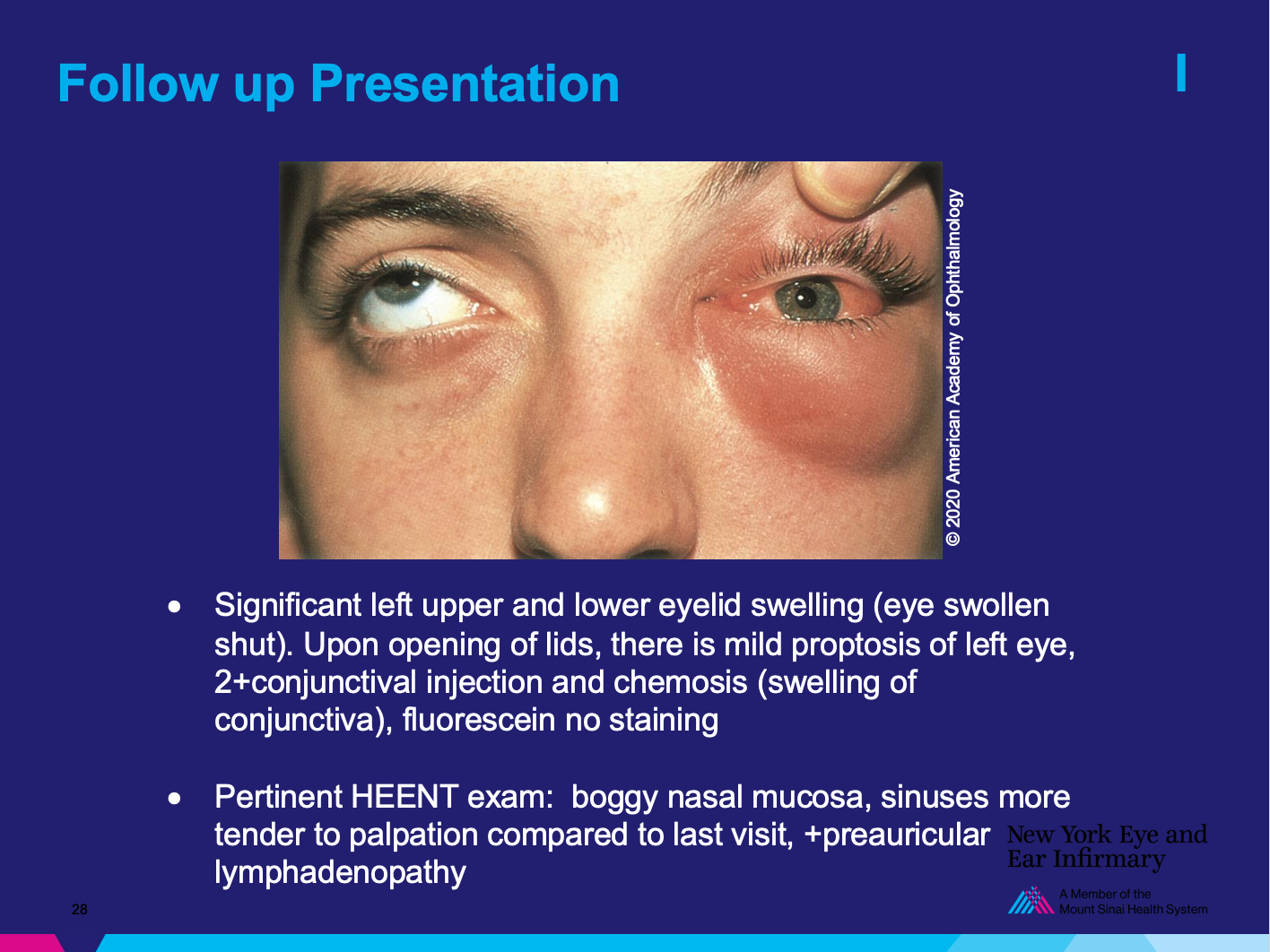


- **Advance** to next slide which shows description, and additional physical exam info

**Pause for discussion:**

- **Ask, “What’s on the differential now?”:** Ask students to type in the top 2-3 on differential diagnoses in chat function.
- *Possible responses:*
  - *Orbital cellulitis*
  - *Thyroid orbitopathy*
  - *Retrobulbar hematoma*
  - *Orbital mass (meningioma)*
- Again, co-preceptor can scribe on “Whiteboard” and Lead preceptor summarizes top items and facilitates discussion
- Review why orbital cellulitis is most likely entity
- Discuss importance of palpating sinuses and looking inside nose as sinus is often source of spread of infection to orbit (**show slide J,** review pertinent orbital anatomy, discuss that ethmoid bone/lamina papyracea is common site through which sinus infection can spread to orbit)


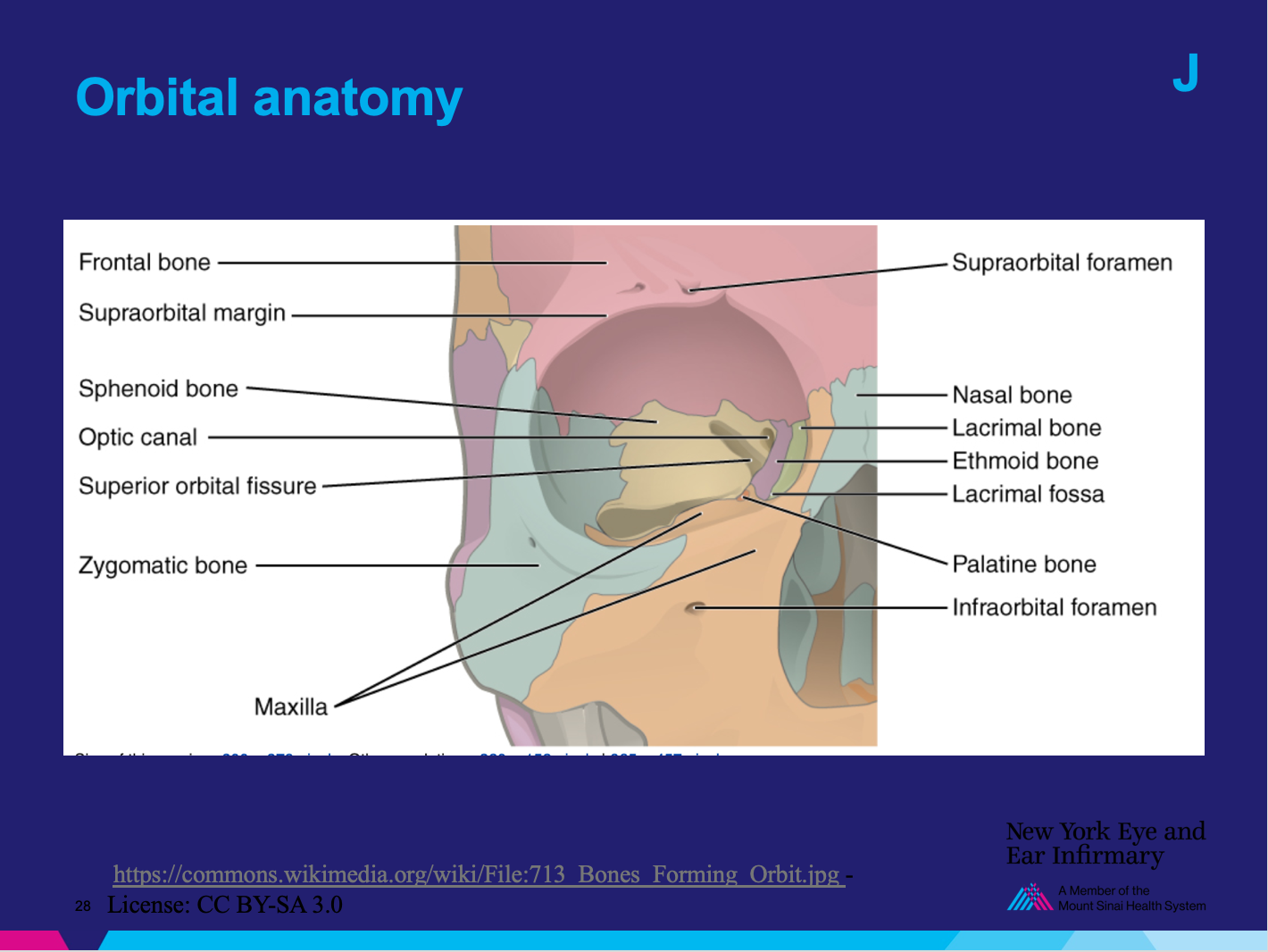


**6. CASE CONCLUSION (5 mins)**

- Patient sent to the emergency department for urgent computed tomography (CT) scan of the orbits **(show slide K)**
- Review representative image which shows left sided sinus opacification, left eye proptosis, and eyelid edema


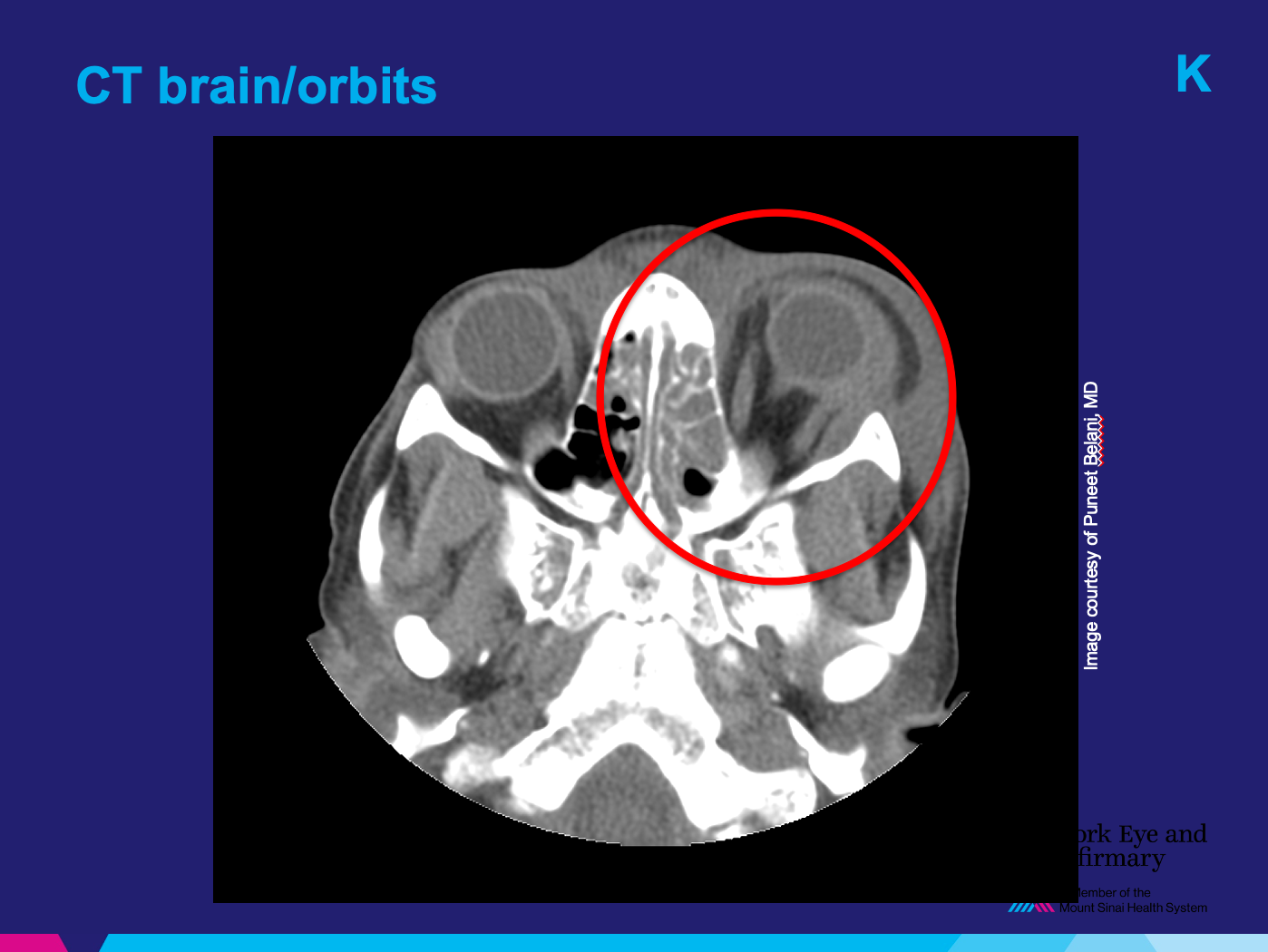


- - Advance to next slide to show case conclusion **(Slide L)**:


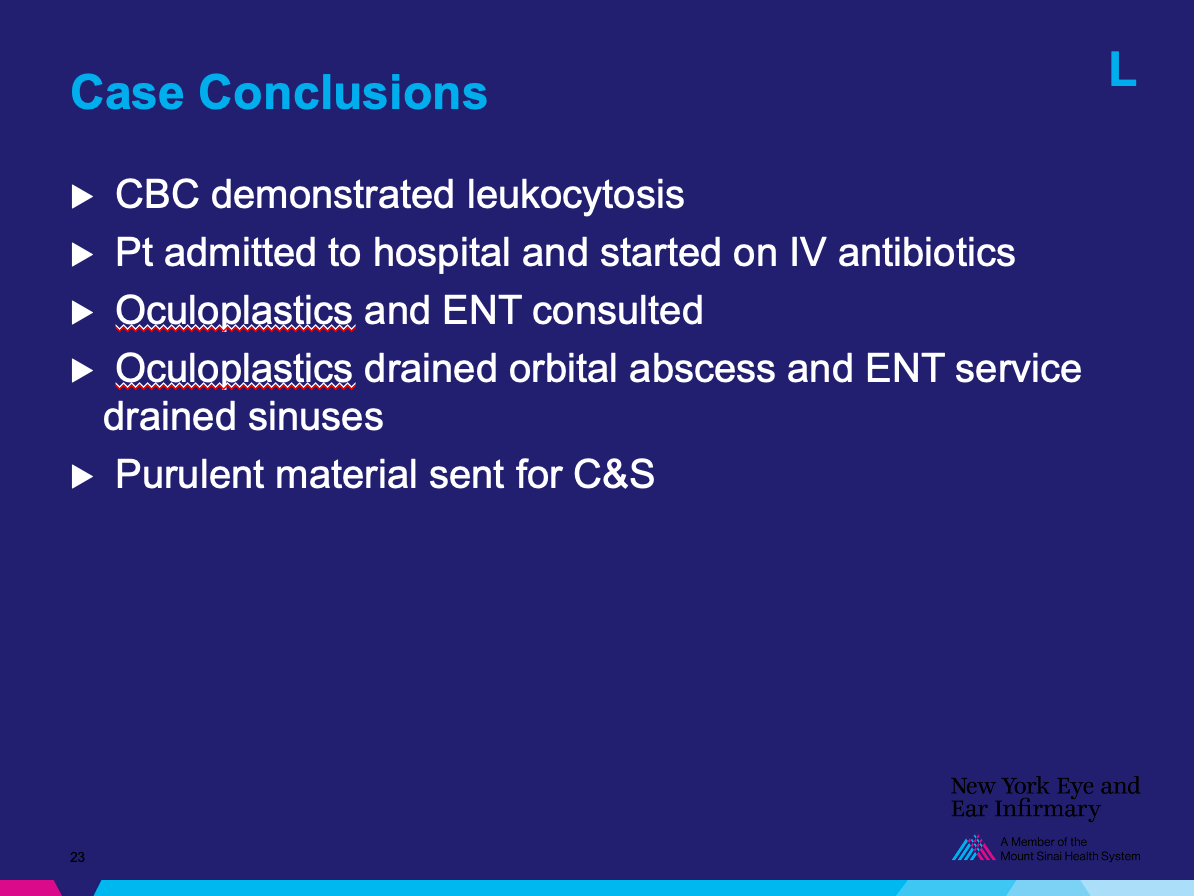


**7. COMPLETE POST-TEST AND REVIEW ANSWERS (10 mins)**

- share post-test link (i.e. in chat function or via emai)
- Review answers (if using online format such as Google Forms, can **“share”** Google Form when reviewing answers)
- **Optional poll #2:** Survey of student perceptions of session
