# Supplementary material for: A Virtual Clinical Reasoning Case for Medical Students Using an Ophthalmology Model: A Case of Red Eye
Source: MedEdPORTAL. 2021 Mar 4;17:11117. doi: 10.15766/mep_2374-8265.11117 (PMC7970637; doi:10.15766/mep_2374-8265.11117)
Supplement: Supplementary file 1 — Faculty Guide.docxPre- and Posttest.docxTemplate for Google Document.docxRed Eye Clinical Reasoning Presentation.pptxRed Eye Session Polls.docx [file mep_2374-8265.11117-s001.zip › B. Pre- and Posttest.docx]

**Red Eye Clinical Reasoning Pre and Post Test**

*(Please note: correct answers are indicated by bold font)*

1. A 75 year old male with atrial fibrillation on coumadin presents complaining of redness in his right eye which he noticed upon awakening this morning. He denies change in vision, pain, or discharge. On external exam you see the following:


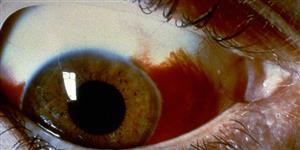


What’s the most likely diagnosis?

1. Episcleritis
2. Scleritis
3. Allergic conjunctivitis
4. **Subconjunctival hemorrhage**
5. A 35 year old female , contact lens wearer, presents complaining of pain and redness in her left eye which began yesterday. She states her vision is slightly blurry but denies discharge or photophobia. She also reports having cold sores around her mouth. She reports good contact lens hygiene and never sleeps in her lenses. On external exam, under cobalt blue light with fluorescein staining, you see the following:


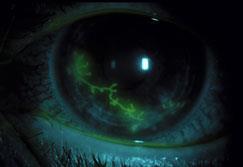


What’s the most likely diagnosis?

1. Corneal ulcer
2. Corneal abrasion
3. **Herpetic dendrite**
4. uveitis
5. A 25 year old male presents complaining of 3 days of redness and discomfort in his right eye. He also reports significant photophobia. His vision is slightly blurry and he denies discharge. He notes one prior episode a few months ago that coincided with some back pain and states both symptoms resolved after a few days on their own. On external exam you see the following:


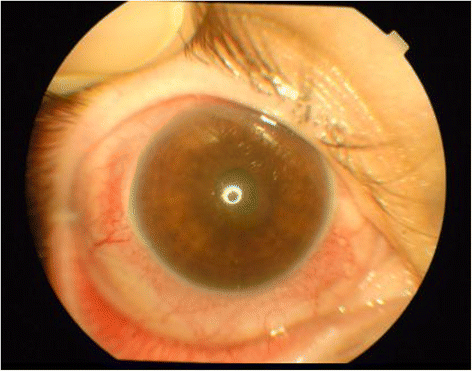


What’s the most likely diagnosis?

1. **Anterior uveitis**
2. Episcleritis
3. Allergic conjunctivitis
4. Viral conjunctivitis
5. A 60 year old female presents complaining of a foreign body sensation, burning, and tearing from both of her eyes for a few weeks. She also has noted crusting around her eyelashes. She states the symptoms are usually worst in the morning and get better throughout the day. She denies change in vision. On external exam you see the following. What is the most likely diagnosis?


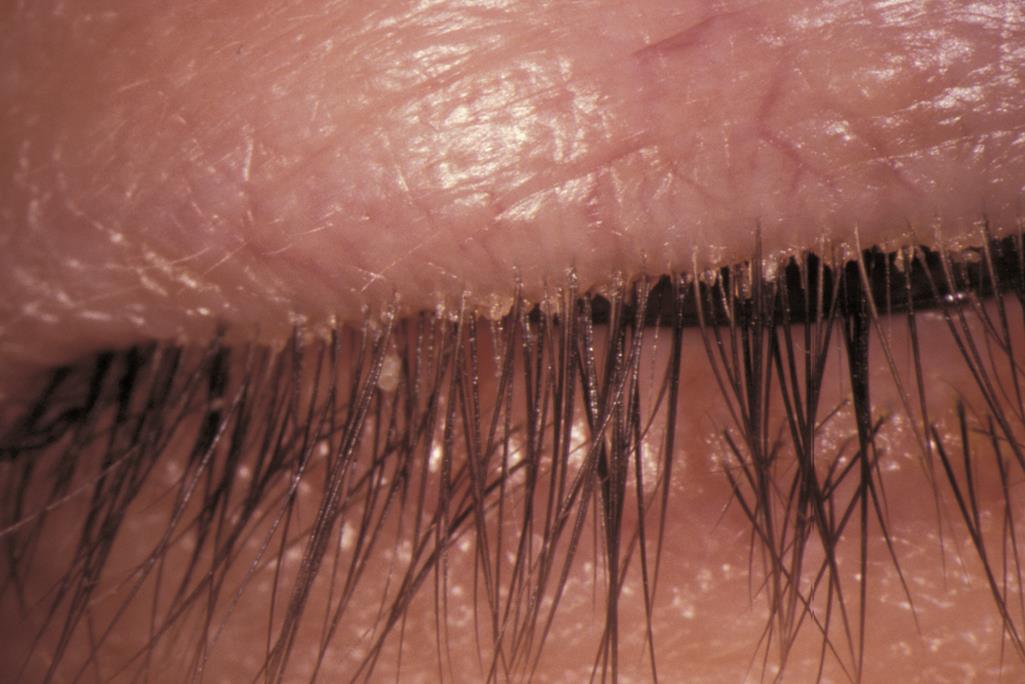


1. Allergic conjunctivitis
2. Chalazion
3. Dry eye syndrome
4. **Blepharitis**
5. A 55 year old female presents complaining of severe left eye pain, redness, headache, and blurred vision. She was at the movie theater when the symptoms began. She is also having severe nausea and vomits during the history. She mentions seeing haloes around lights. Her eye is firm to palpation. On external exam you see the following:


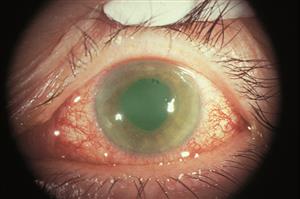


What’s the most likely diagnosis?

1. Scleritis
2. **Acute angle closure glaucoma**
3. Subconjunctival hemorrhage
4. Thyroid related eye disease

**Image attribution:**

1. Image retrieved from: <https://www.aao.org/eye-health/diseases/what-is-subconjunctival-hemorrhage> on June 5, 2020. Permission received from the American Academy of Ophthalmology.
2. Image retrieved from: <https://www.aao.org/eye-health/diseases/herpes-keratitis-cause> on June 5, 2020. Permission received from the American Academy of Ophthalmology.
3. Image by Rahmi Duman, retrieved from: <https://commons.wikimedia.org/wiki/File:Fig-1-Photograph-showed-non-granulomatous-anterior-uveitis-with-nuclear-cataract-and-ciliary-injection-in-the-right-eye.gif> on June 1, 2020. Creative Commons license associated: <https://creativecommons.org/licenses/by/4.0/deed.en>.
4. Image retrieved from: [https://www.aao.org/preferred-practice-pattern/blepharitis](https://dcsmsvpn.mssm.edu/f5-w-68747470733a2f2f75726c646566656e73652e70726f6f66706f696e742e636f6d$$/v2/url?u=https-3A__www.aao.org_preferred-2Dpractice-2Dpattern_blepharitis&d=DwMFaQ&c=shNJtf5dKgNcPZ6Yh64b-A&r=y84hFoVA-WTejA5LKM1e74s9hLSa7jnLt7FuyfDKK7Q&m=w21x6ftMpOhLVnOzpmYbiq-d_uhP2sSCgw71jRBR9S0&s=Bfa1-xRo5QnYr3R_ibMCo6fnXBLTUhuoKMi6GRDIZxk&e=) on June 5, 2020. Permission received from the American Academy of Ophthalmology.
5. Image retrieved from: <https://www.aao.org/disease-review/abnormalities-associated-with-closed-angle> on June 5, 2020. Permission received from the American Academy of Ophthalmology.
