# Supplementary material for: A Virtual Clinical Reasoning Case for Medical Students Using an Ophthalmology Model: A Case of Red Eye
Source: MedEdPORTAL. 2021 Mar 4;17:11117. doi: 10.15766/mep_2374-8265.11117 (PMC7970637; doi:10.15766/mep_2374-8265.11117)
Supplement: Supplementary file 1 — Faculty Guide.docxPre- and Posttest.docxTemplate for Google Document.docxRed Eye Clinical Reasoning Presentation.pptxRed Eye Session Polls.docx [file mep_2374-8265.11117-s001.zip › C. Template for Google Document.docx]

Template for Google Document:

1. Write up an example History of the Present Illness (HPI) for your assigned entity (possible chief complaint, presenting history and associated symptoms)
2. Find a representative image and paste it under HPI

**Red Eye Differential Diagnosis:**

1. Acute angle closure glaucoma
2. Dry eye syndrome
3. Scleritis
4. Viral conjunctivitis
5. Pterygium
6. Corneal ulcer
7. Corneal abrasion
8. Subconjunctival hemorrhage
9. Uveitis
10. Blepharitis
