# Supplementary material for: A Virtual Clinical Reasoning Case for Medical Students Using an Ophthalmology Model: A Case of Red Eye
Source: MedEdPORTAL. 2021 Mar 4;17:11117. doi: 10.15766/mep_2374-8265.11117 (PMC7970637; doi:10.15766/mep_2374-8265.11117)
Supplement: Supplementary file 1 — Faculty Guide.docxPre- and Posttest.docxTemplate for Google Document.docxRed Eye Clinical Reasoning Presentation.pptxRed Eye Session Polls.docx [file mep_2374-8265.11117-s001.zip › D. Red Eye Clinical Reasoning Presentation.pptx]

## Slide 1
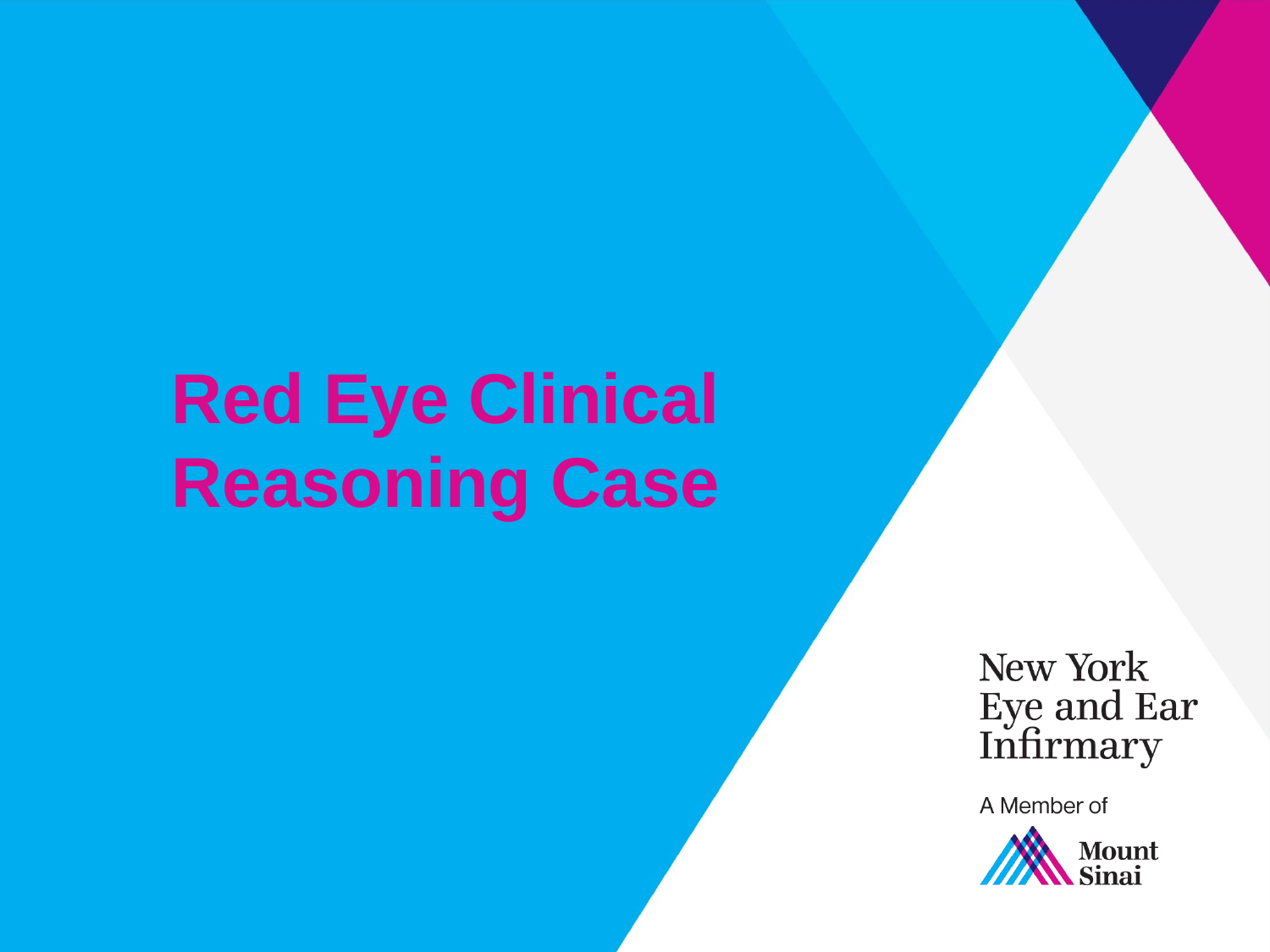

# Red Eye Clinical Reasoning Case

## Slide 2
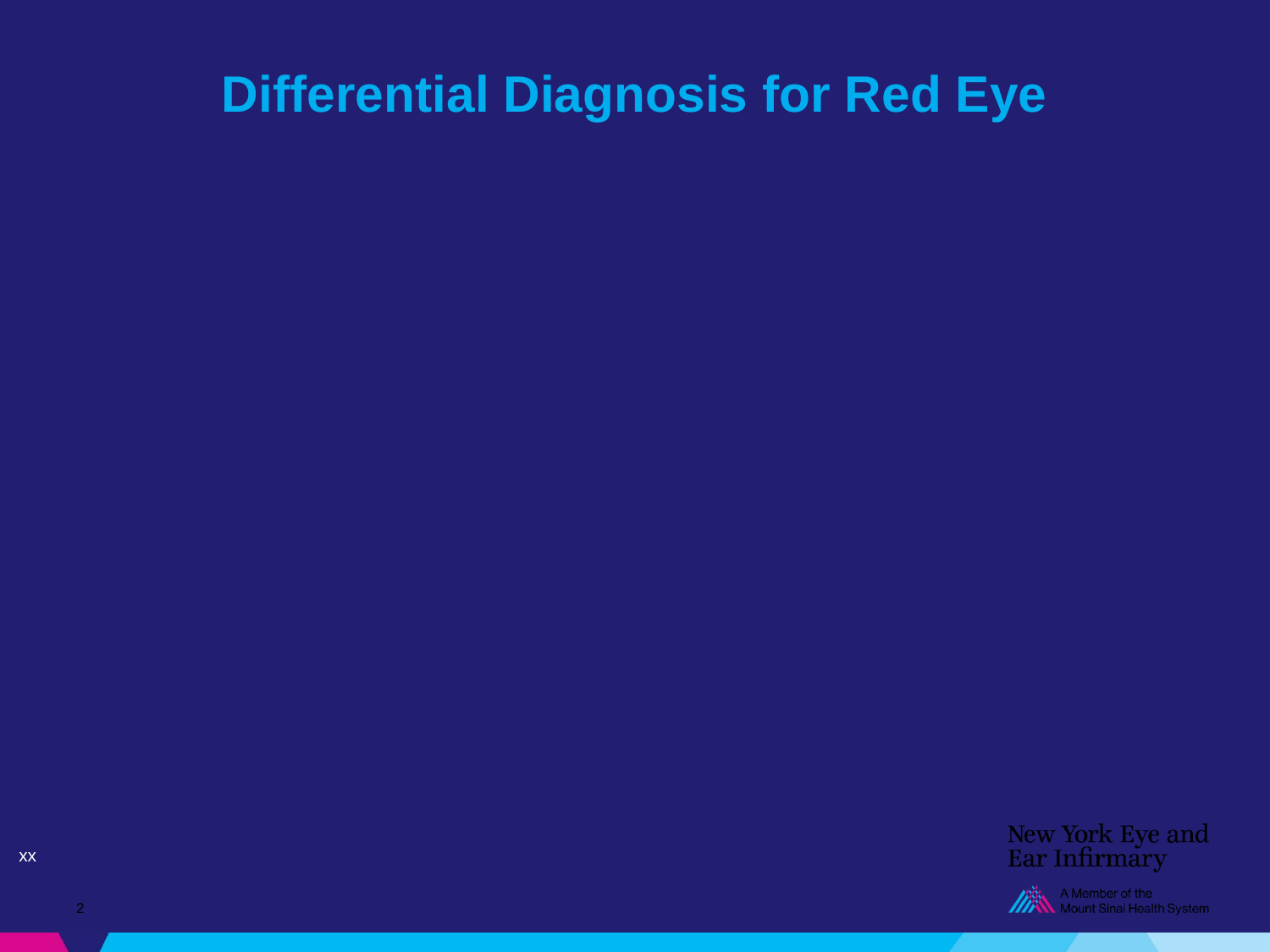

# Differential Diagnosis for Red Eye
xx
2

## Slide 3
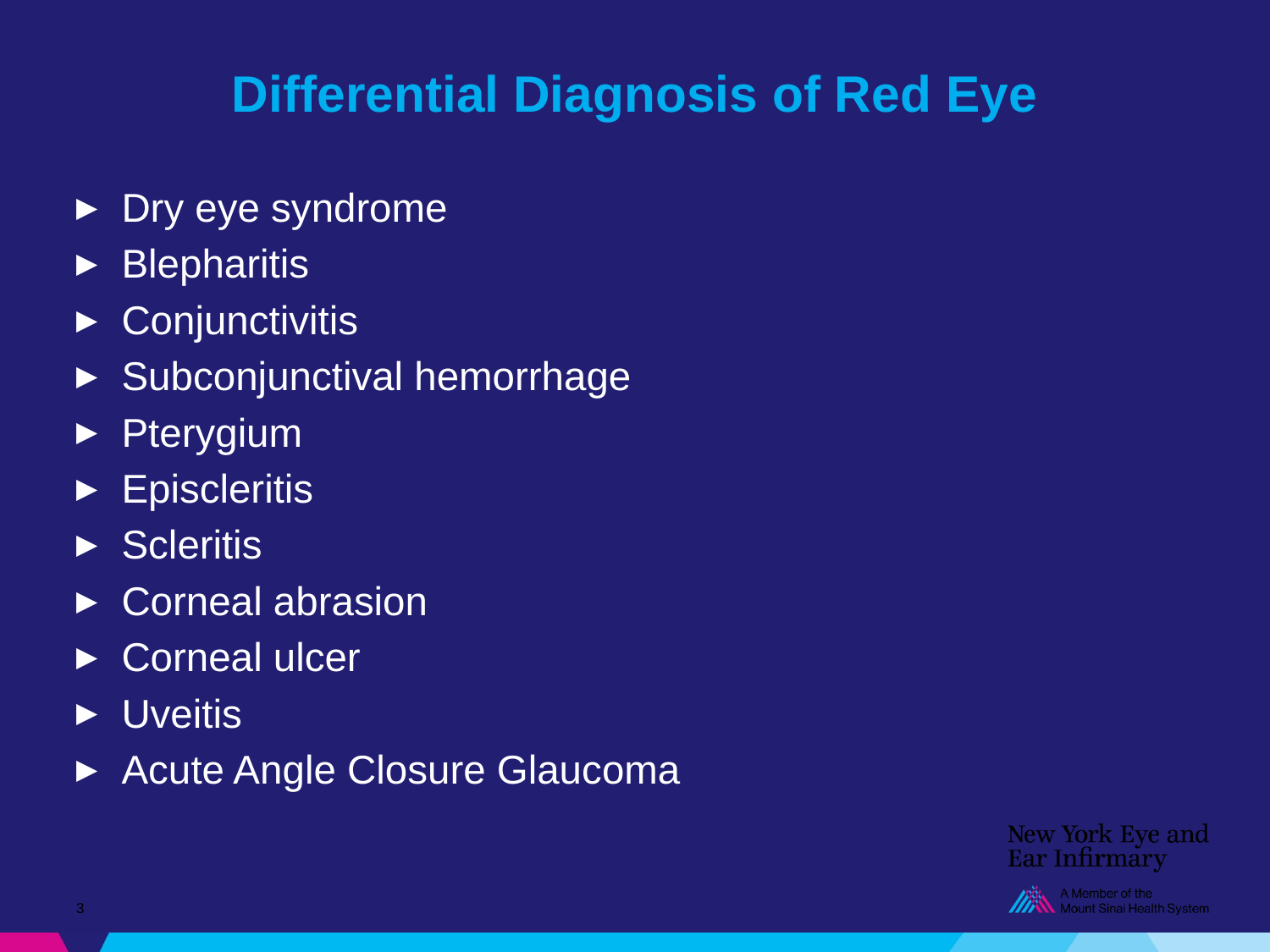

# Differential Diagnosis of Red Eye
Dry eye syndrome
Blepharitis
Conjunctivitis
Subconjunctival hemorrhage
Pterygium
Episcleritis
Scleritis
Corneal abrasion
Corneal ulcer
Uveitis
Acute Angle Closure Glaucoma
3

## Slide 4
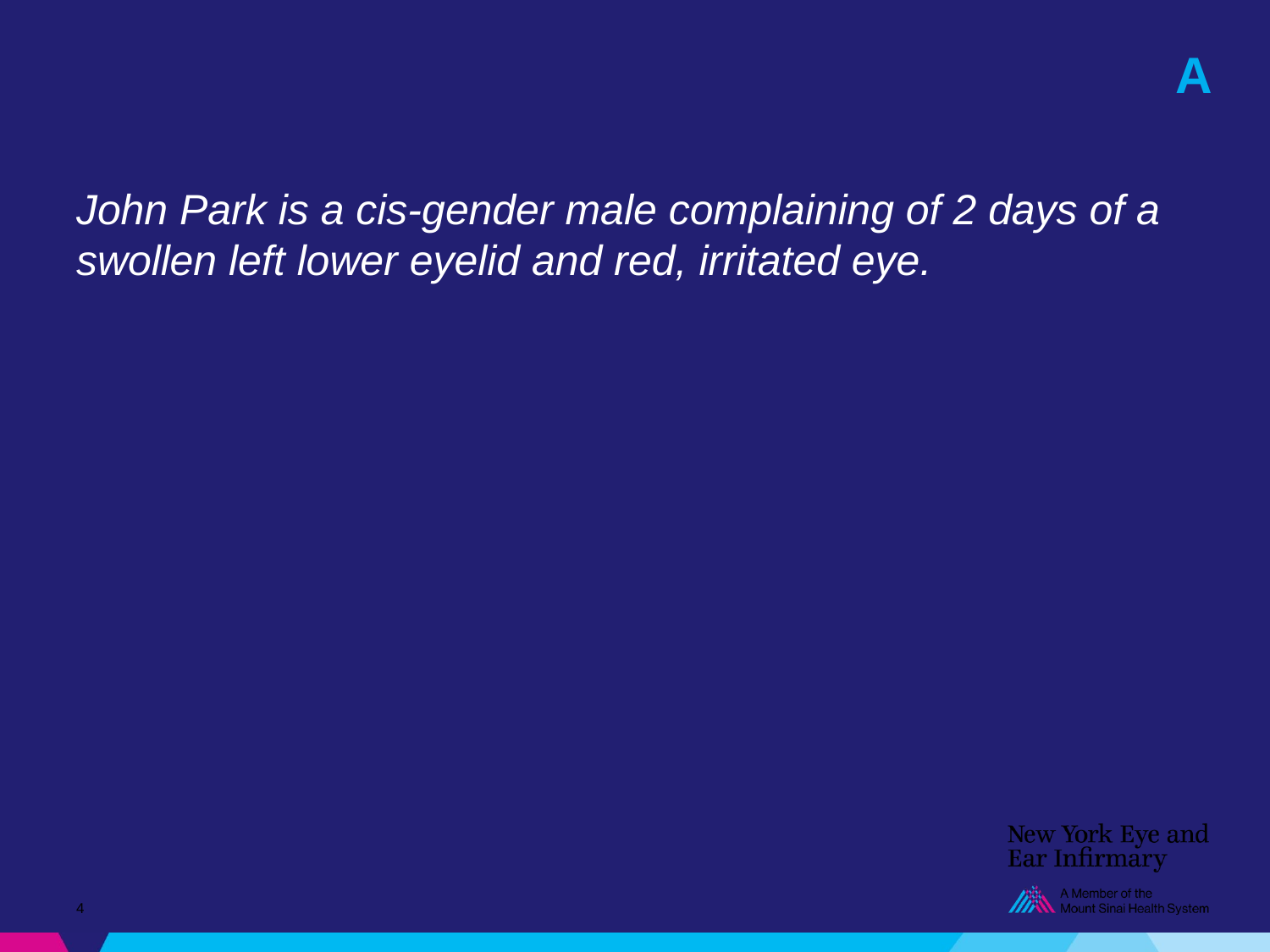

# A
John Park is a cis-gender male complaining of 2 days of a swollen left lower eyelid and red, irritated eye.
4

## Slide 5
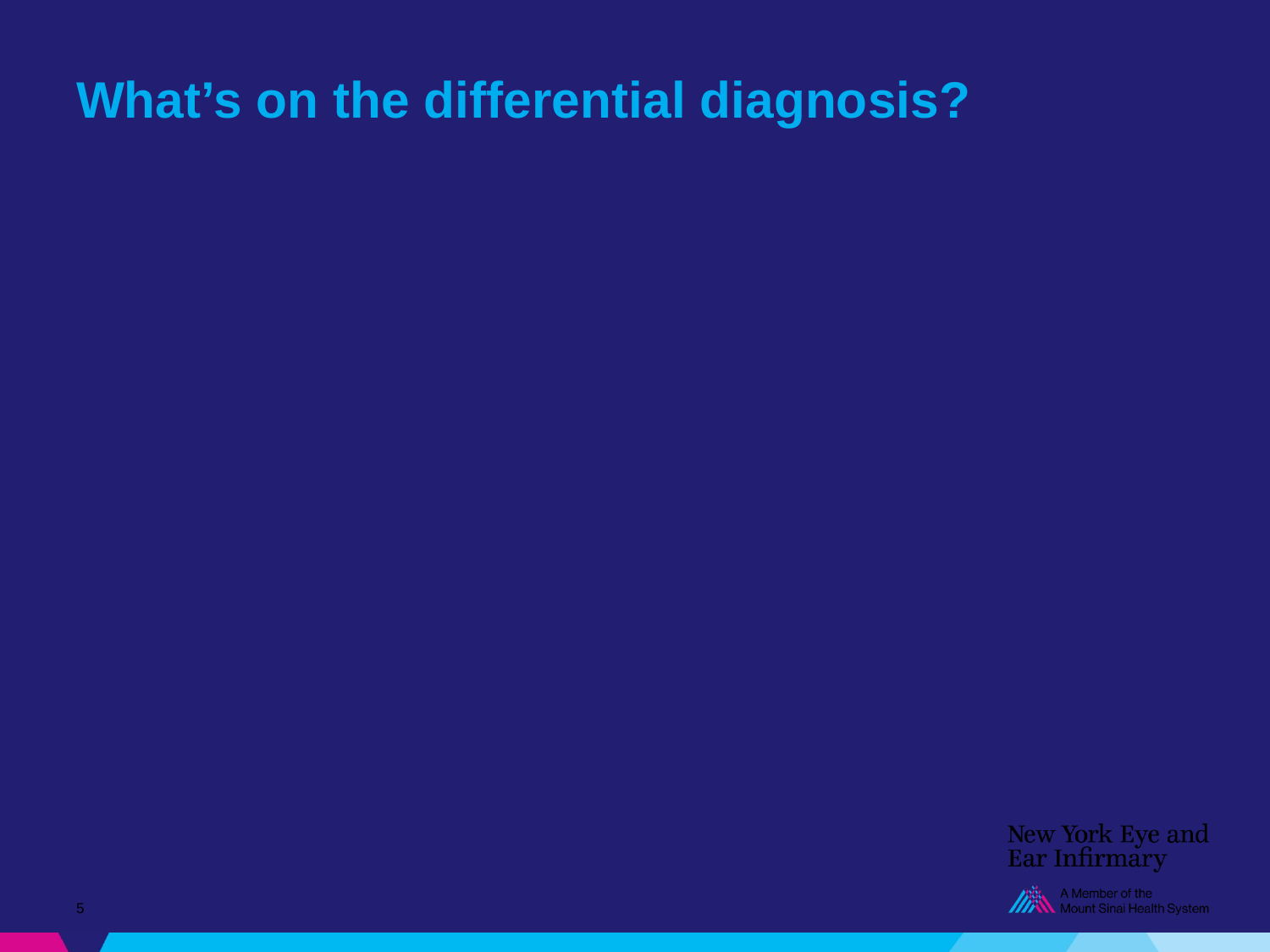

# What’s on the differential diagnosis?
5

## Slide 6
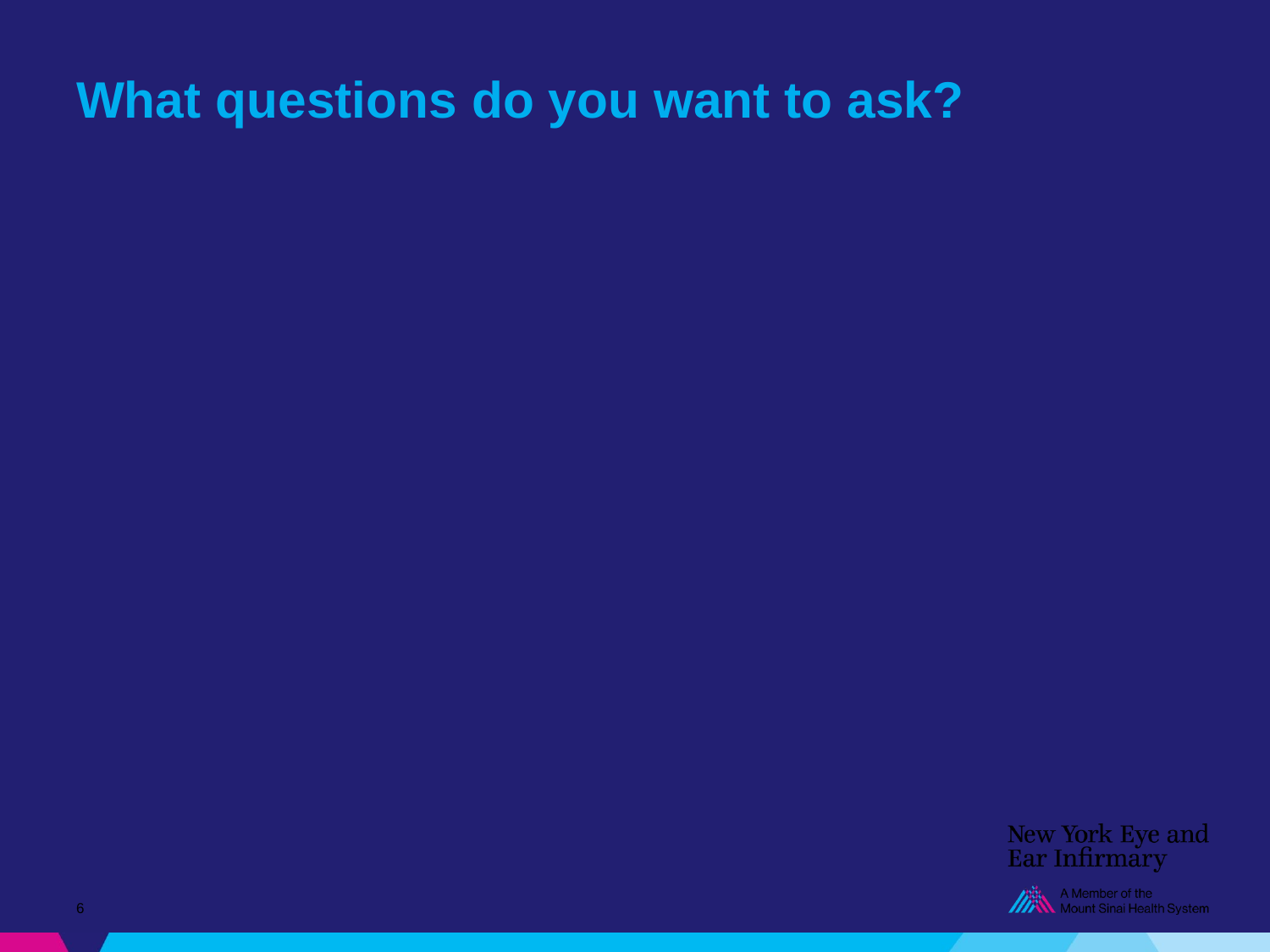

# What questions do you want to ask?
6

## Slide 7
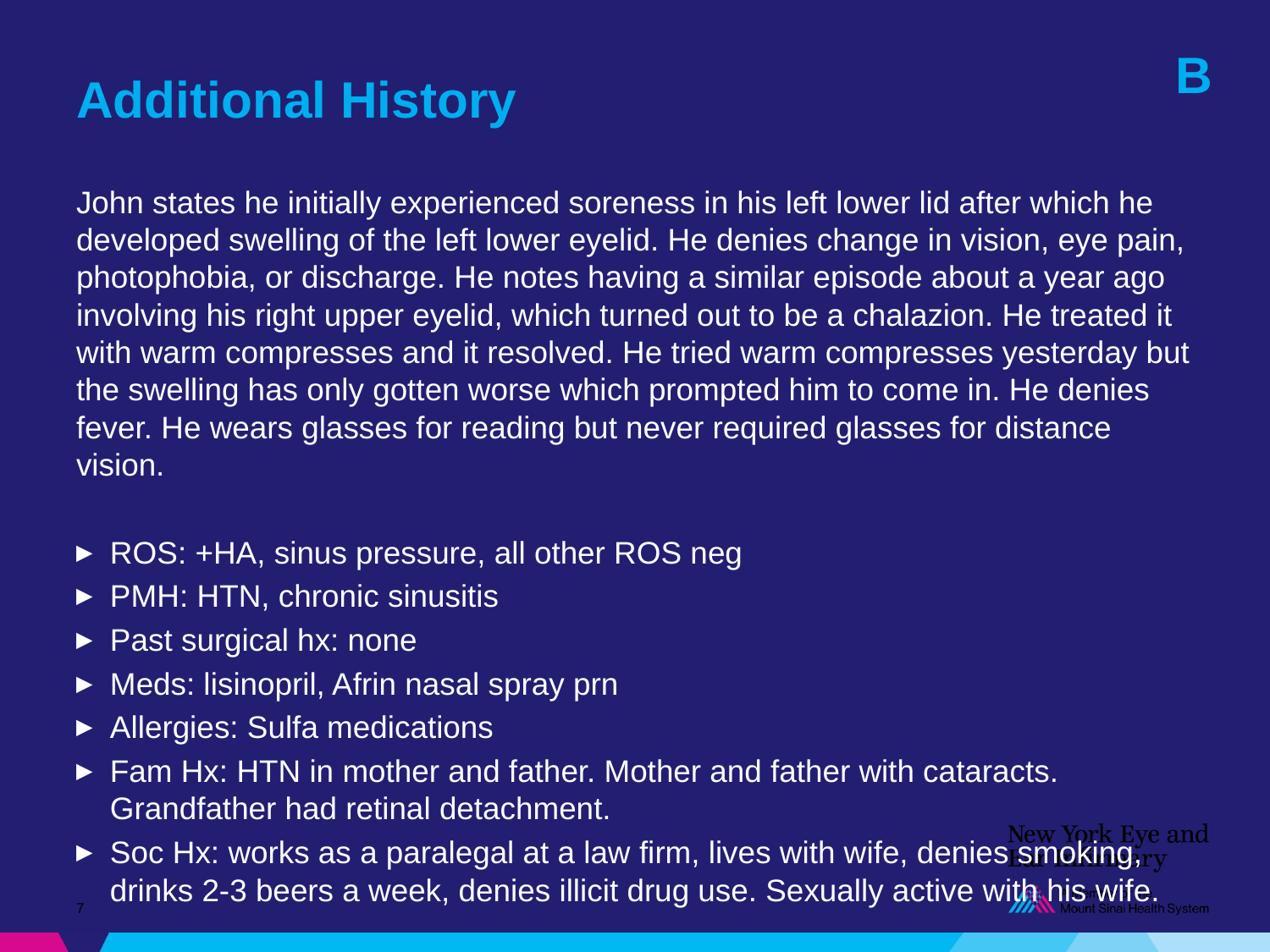

B
# Additional History
John states he initially experienced soreness in his left lower lid after which he developed swelling of the left lower eyelid. He denies change in vision, eye pain, photophobia, or discharge. He notes having a similar episode about a year ago involving his right upper eyelid, which turned out to be a chalazion. He treated it with warm compresses and it resolved. He tried warm compresses yesterday but the swelling has only gotten worse which prompted him to come in. He denies fever. He wears glasses for reading but never required glasses for distance vision.
ROS: +HA, sinus pressure, all other ROS neg
PMH: HTN, chronic sinusitis
Past surgical hx: none
Meds: lisinopril, Afrin nasal spray prn
Allergies: Sulfa medications
Fam Hx: HTN in mother and father. Mother and father with cataracts. Grandfather had retinal detachment.
Soc Hx: works as a paralegal at a law firm, lives with wife, denies smoking, drinks 2-3 beers a week, denies illicit drug use. Sexually active with his wife.
7

## Slide 8
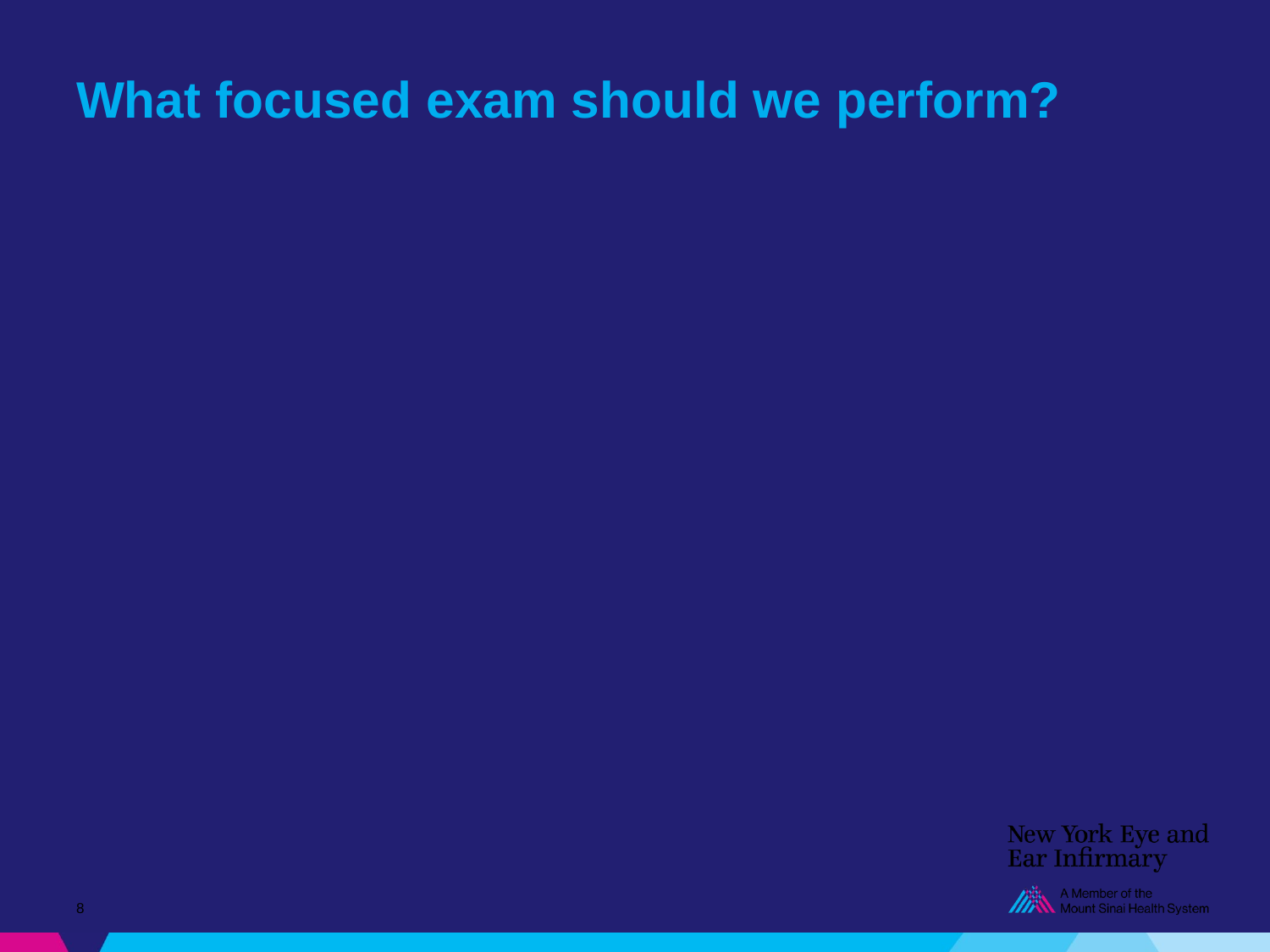

# What focused exam should we perform?
8

## Slide 9
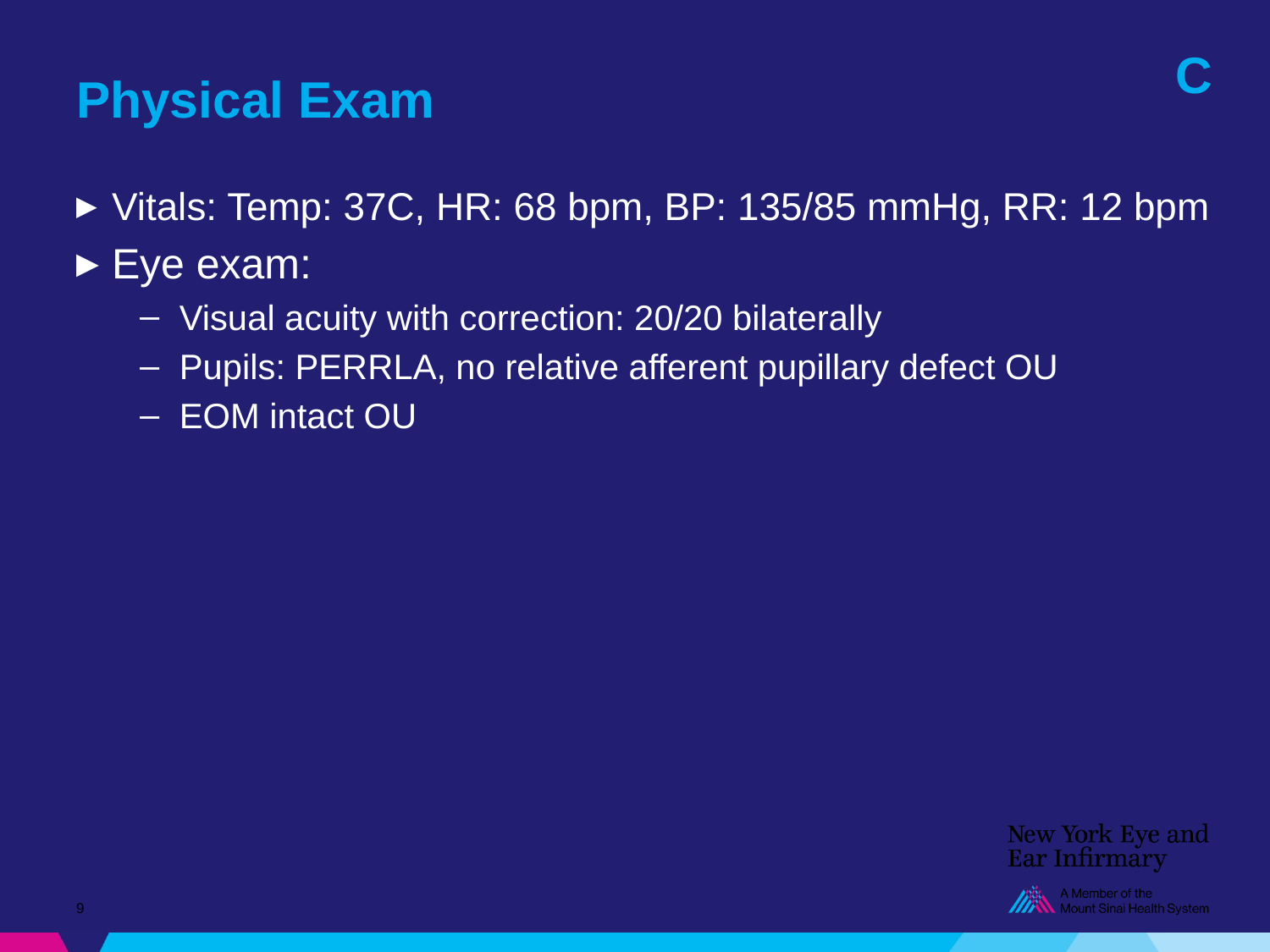

C
# Physical Exam
Vitals: Temp: 37C, HR: 68 bpm, BP: 135/85 mmHg, RR: 12 bpm
Eye exam:
Visual acuity with correction: 20/20 bilaterally
Pupils: PERRLA, no relative afferent pupillary defect OU
EOM intact OU
9

## Slide 10
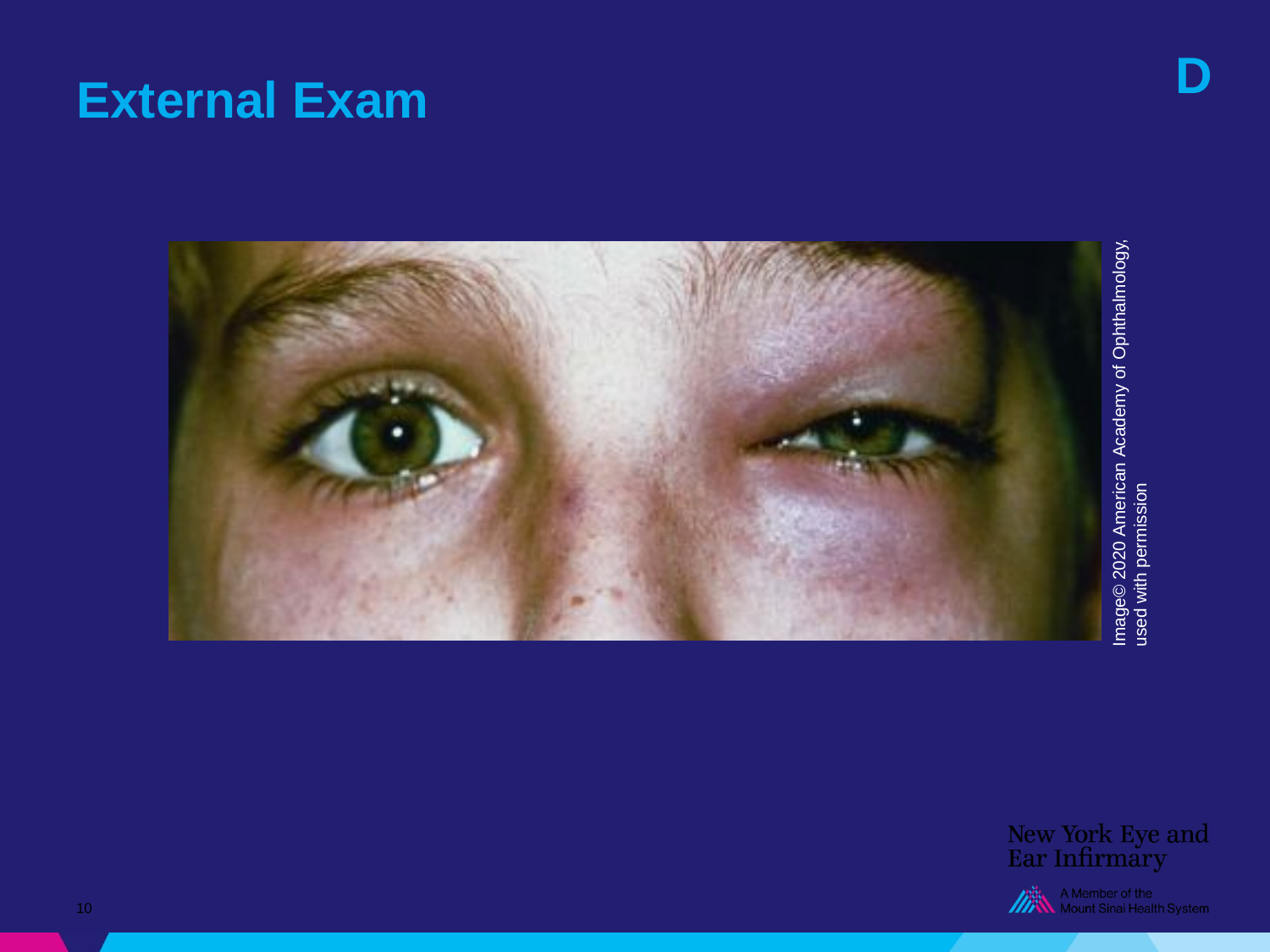

D
# External Exam
Image© 2020 American Academy of Ophthalmology, used with permission
10

## Slide 11
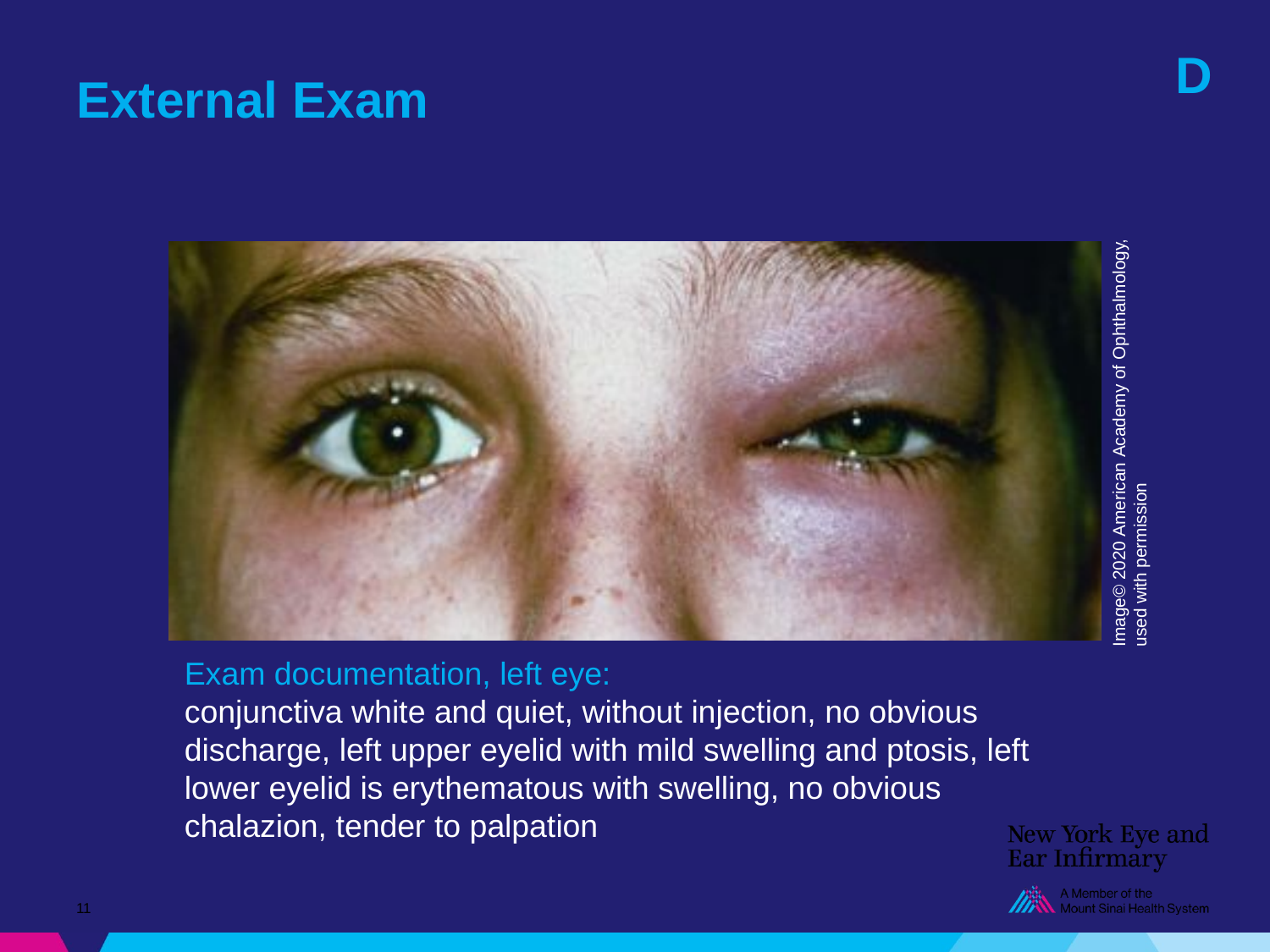

D
# External Exam
Image© 2020 American Academy of Ophthalmology, used with permission
Exam documentation, left eye:
conjunctiva white and quiet, without injection, no obvious discharge, left upper eyelid with mild swelling and ptosis, left lower eyelid is erythematous with swelling, no obvious chalazion, tender to palpation
11

## Slide 12
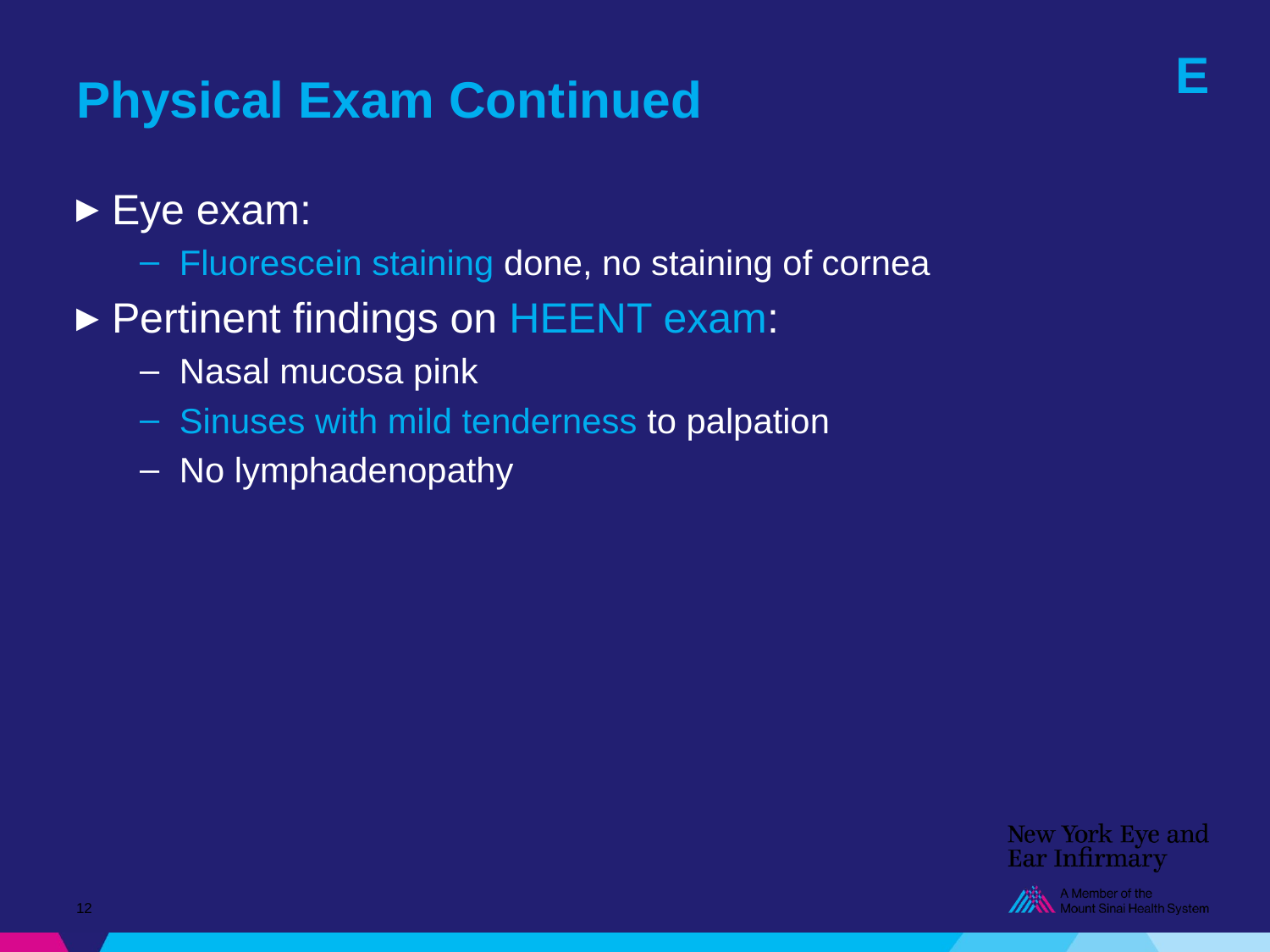

E
# Physical Exam Continued
Eye exam:
Fluorescein staining done, no staining of cornea
Pertinent findings on HEENT exam:
Nasal mucosa pink
Sinuses with mild tenderness to palpation
No lymphadenopathy
12

## Slide 13
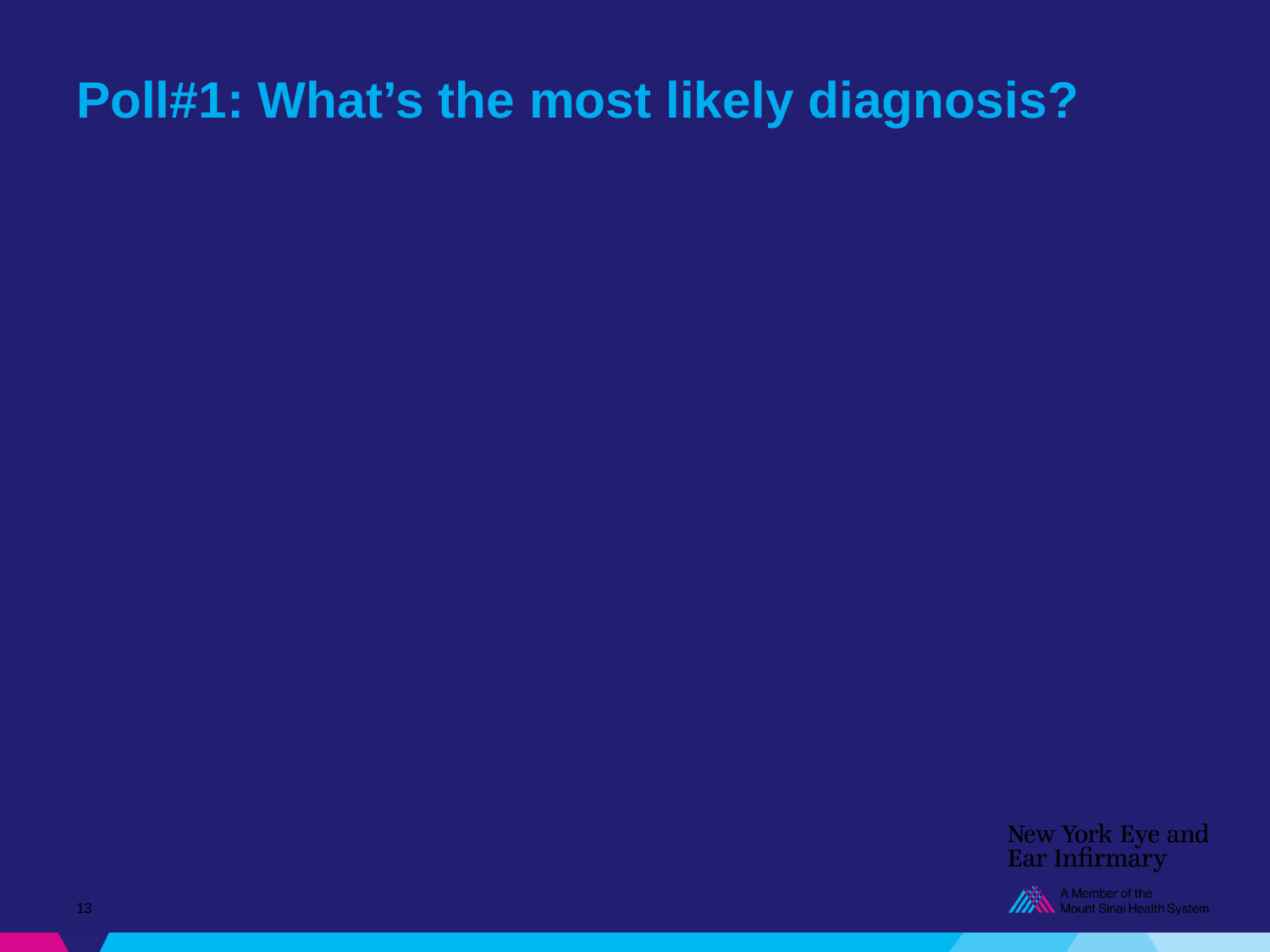

# Poll#1: What’s the most likely diagnosis?
13

## Slide 14
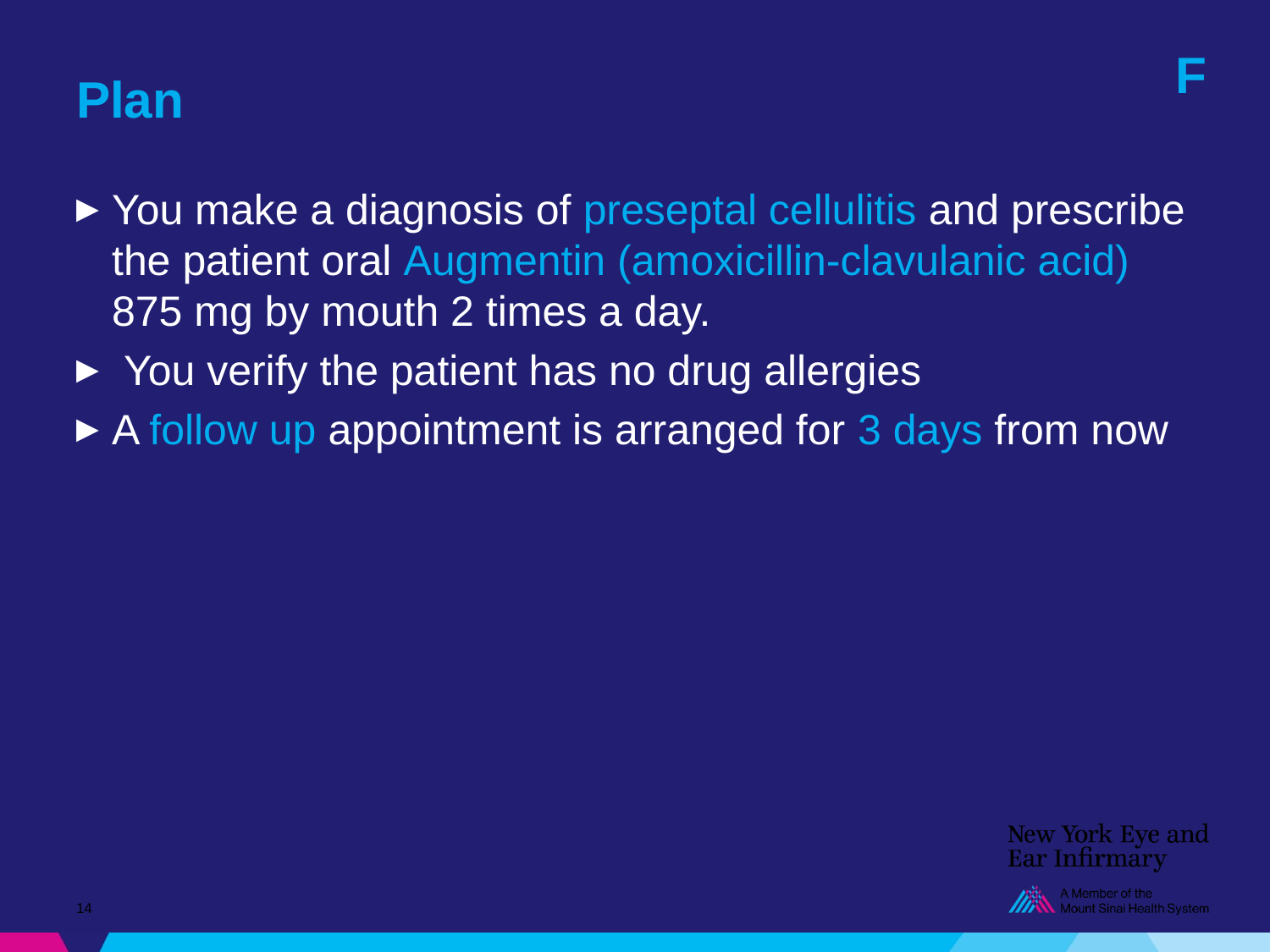

F
# Plan
You make a diagnosis of preseptal cellulitis and prescribe the patient oral Augmentin (amoxicillin-clavulanic acid) 875 mg by mouth 2 times a day.
 You verify the patient has no drug allergies
A follow up appointment is arranged for 3 days from now
14

## Slide 15
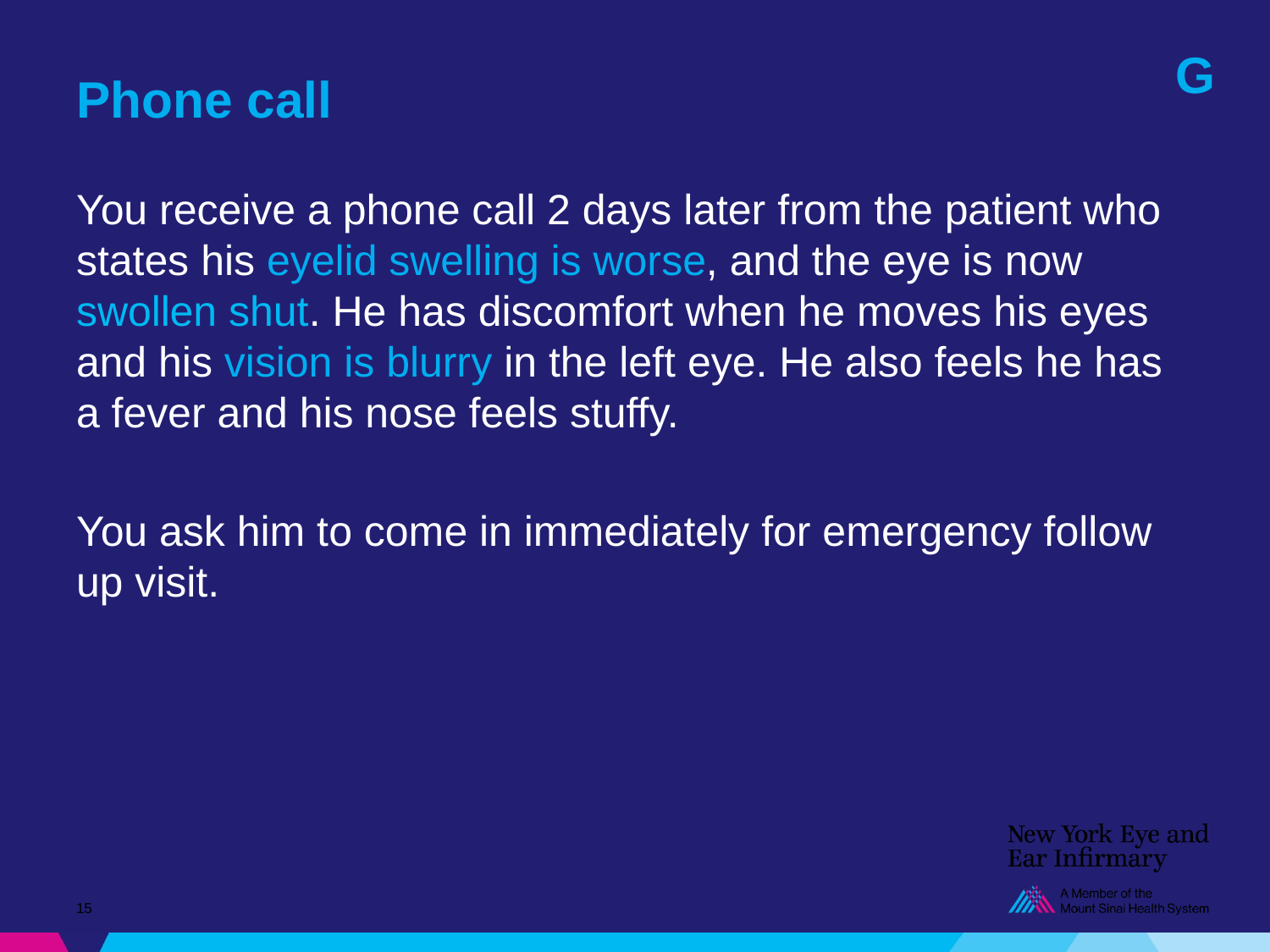

G
# Phone call
You receive a phone call 2 days later from the patient who states his eyelid swelling is worse, and the eye is now swollen shut. He has discomfort when he moves his eyes and his vision is blurry in the left eye. He also feels he has a fever and his nose feels stuffy.
You ask him to come in immediately for emergency follow up visit.
15

## Slide 16
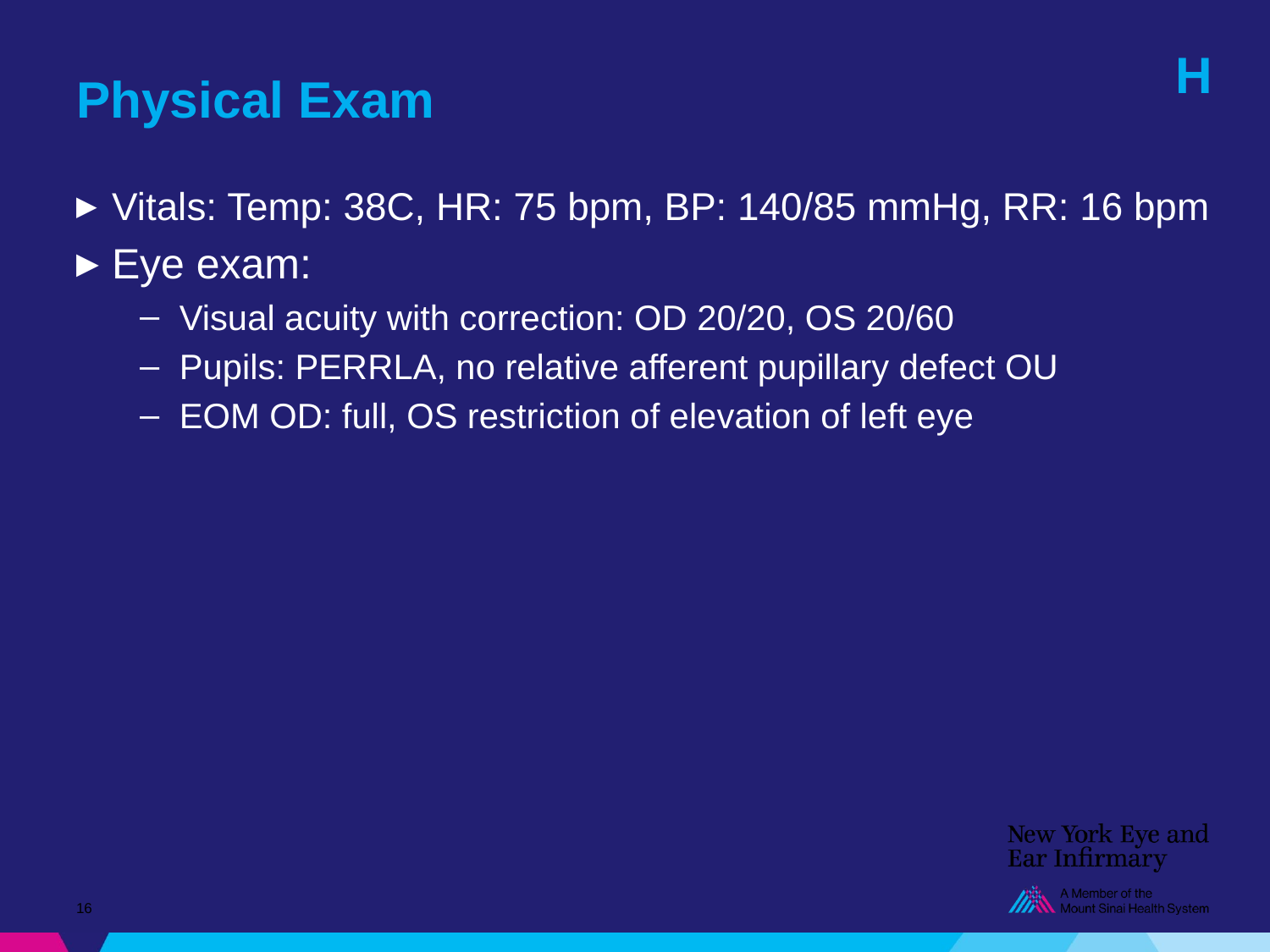

H
# Physical Exam
Vitals: Temp: 38C, HR: 75 bpm, BP: 140/85 mmHg, RR: 16 bpm
Eye exam:
Visual acuity with correction: OD 20/20, OS 20/60
Pupils: PERRLA, no relative afferent pupillary defect OU
EOM OD: full, OS restriction of elevation of left eye
16

## Slide 17
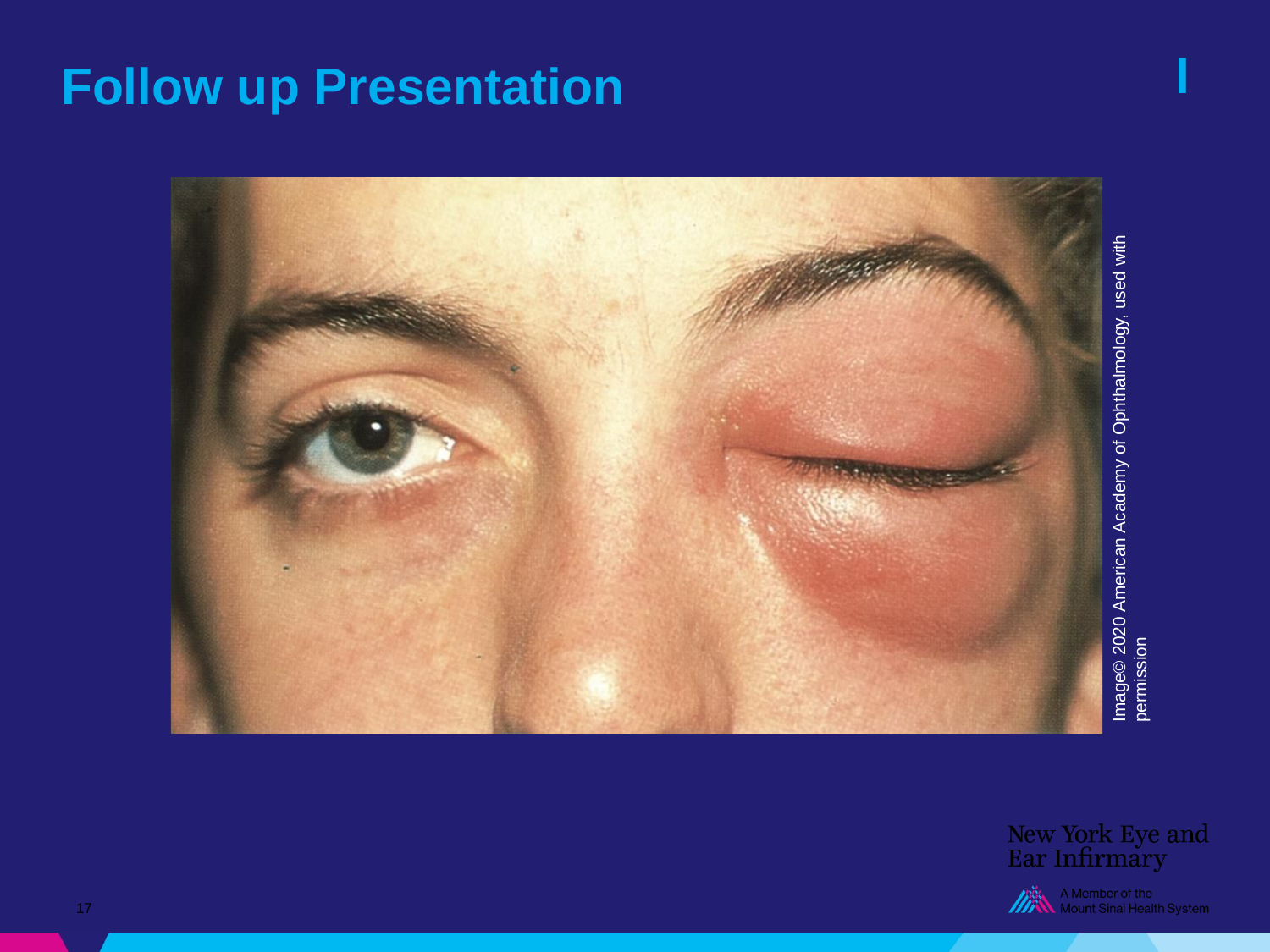

I
# Follow up Presentation
Image© 2020 American Academy of Ophthalmology, used with permission
17

## Slide 18
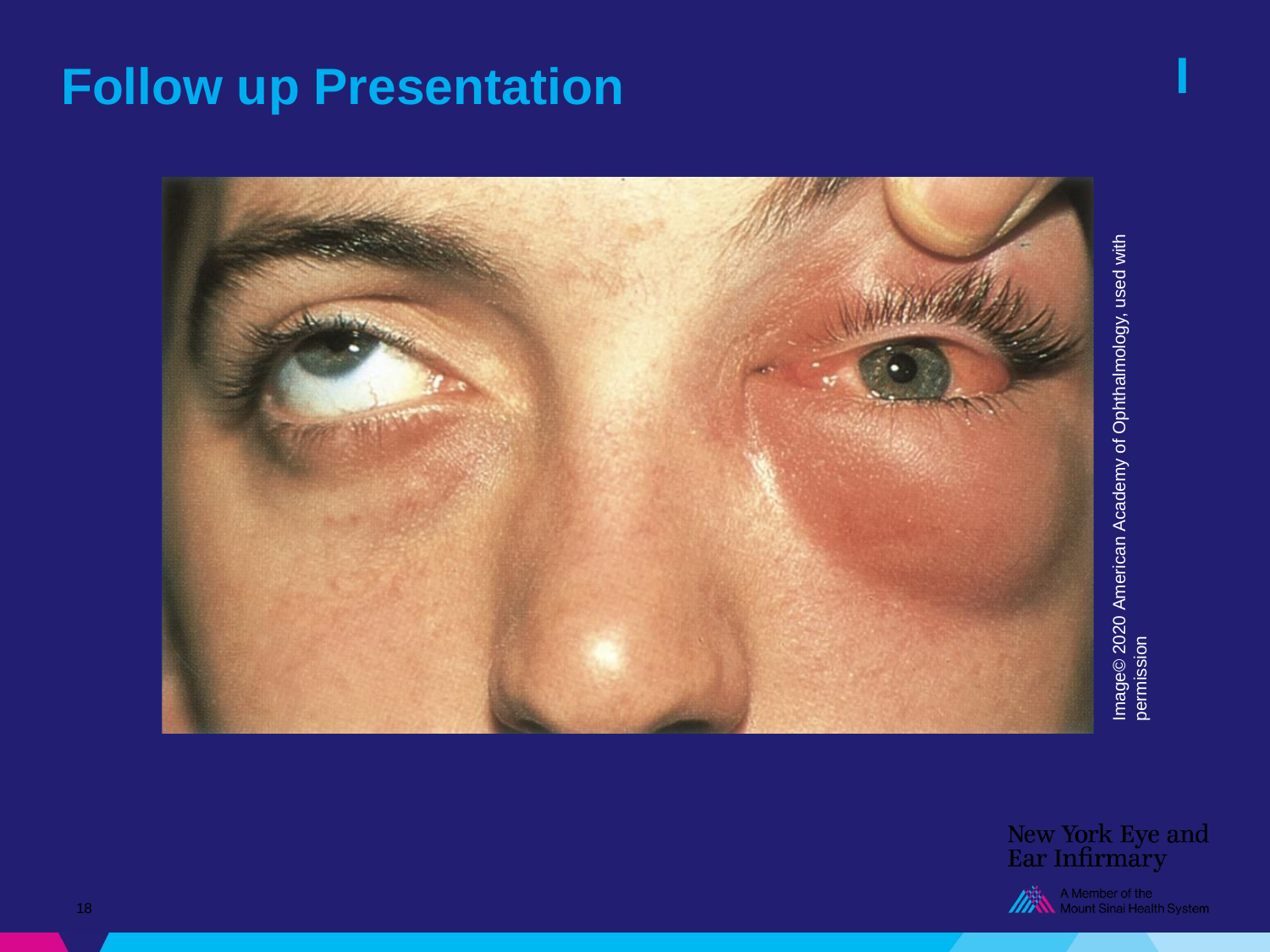

I
# Follow up Presentation
Image© 2020 American Academy of Ophthalmology, used with permission
18

## Slide 19
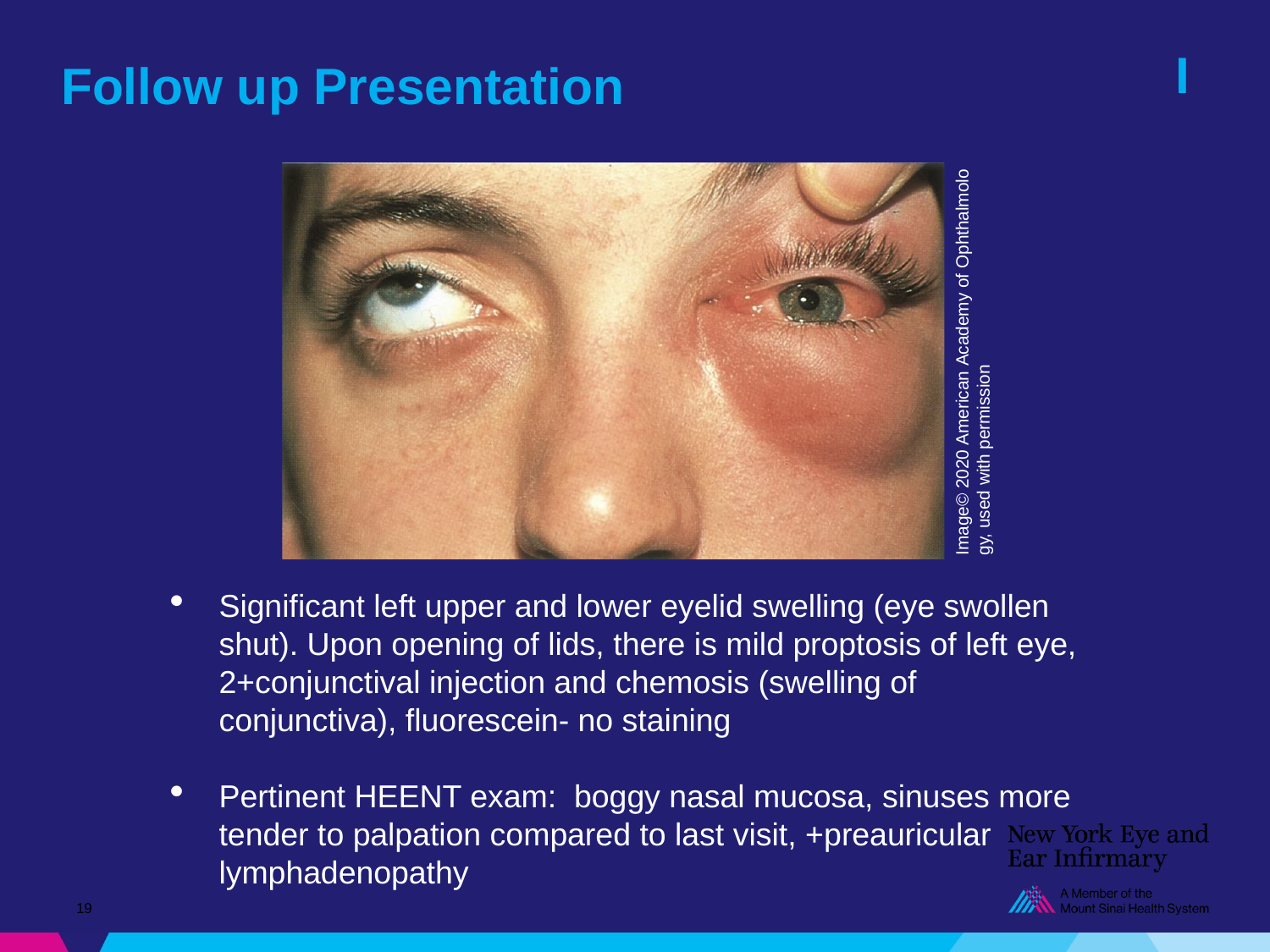

I
# Follow up Presentation
Image© 2020 American Academy of Ophthalmology, used with permission
Significant left upper and lower eyelid swelling (eye swollen shut). Upon opening of lids, there is mild proptosis of left eye, 2+conjunctival injection and chemosis (swelling of conjunctiva), fluorescein- no staining
Pertinent HEENT exam: boggy nasal mucosa, sinuses more tender to palpation compared to last visit, +preauricular lymphadenopathy
19

## Slide 20
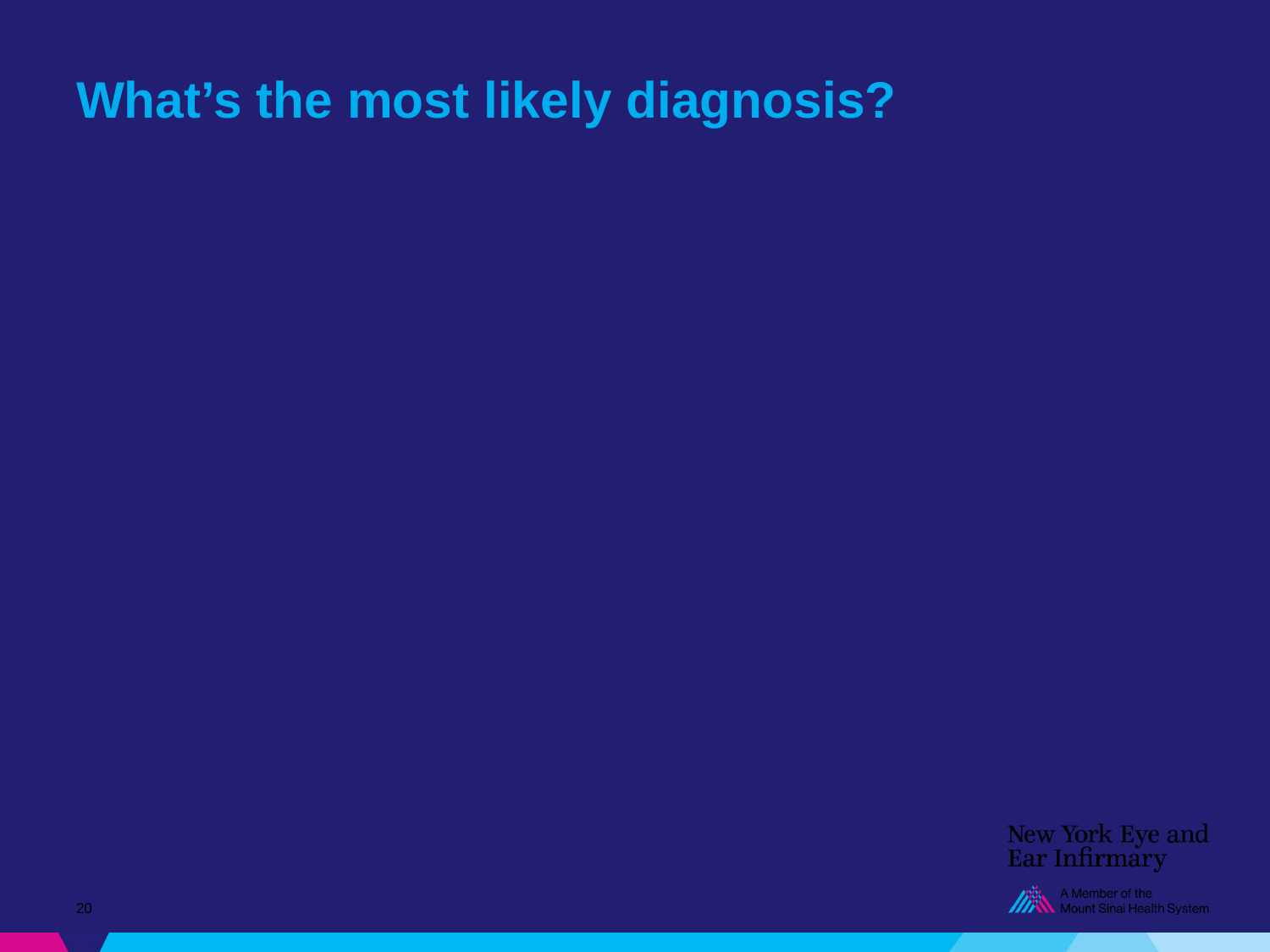

# What’s the most likely diagnosis?
20

## Slide 21
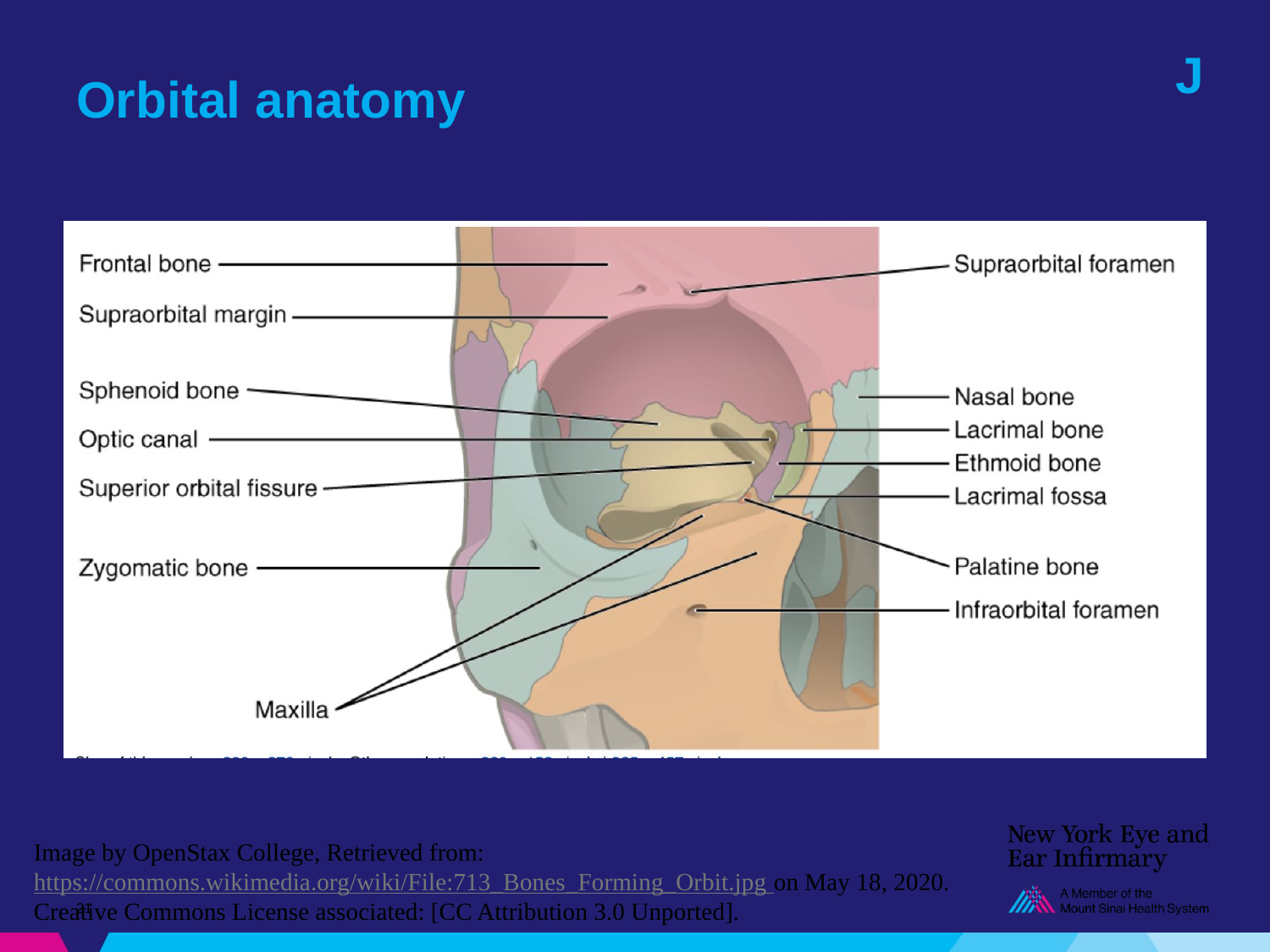

J
# Orbital anatomy
Image by OpenStax College, Retrieved from: https://commons.wikimedia.org/wiki/File:713_Bones_Forming_Orbit.jpg on May 18, 2020. Creative Commons License associated: [CC Attribution 3.0 Unported].
21

## Slide 22
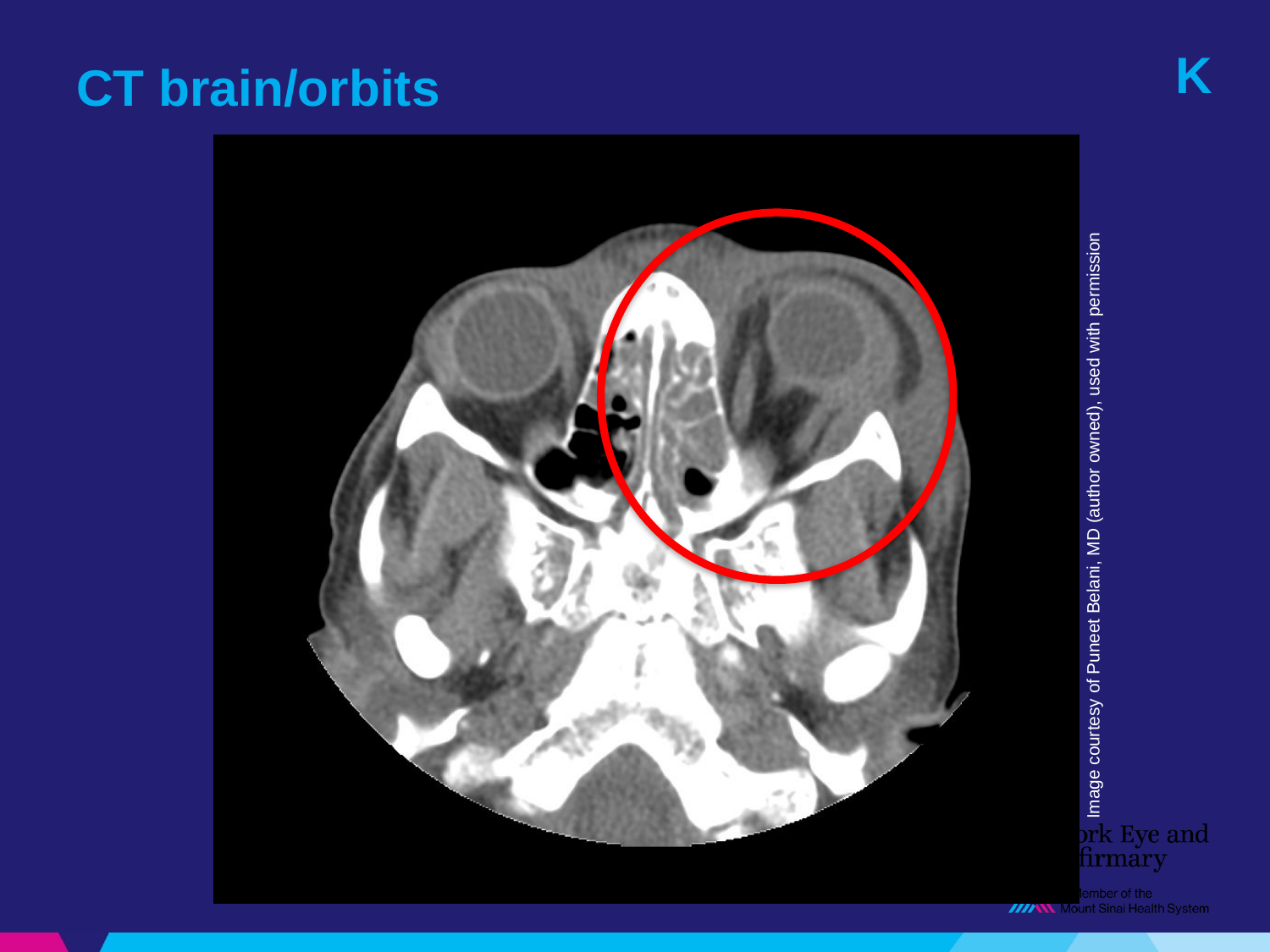

K
# CT brain/orbits
Image courtesy of Puneet Belani, MD (author owned), used with permission

## Slide 23
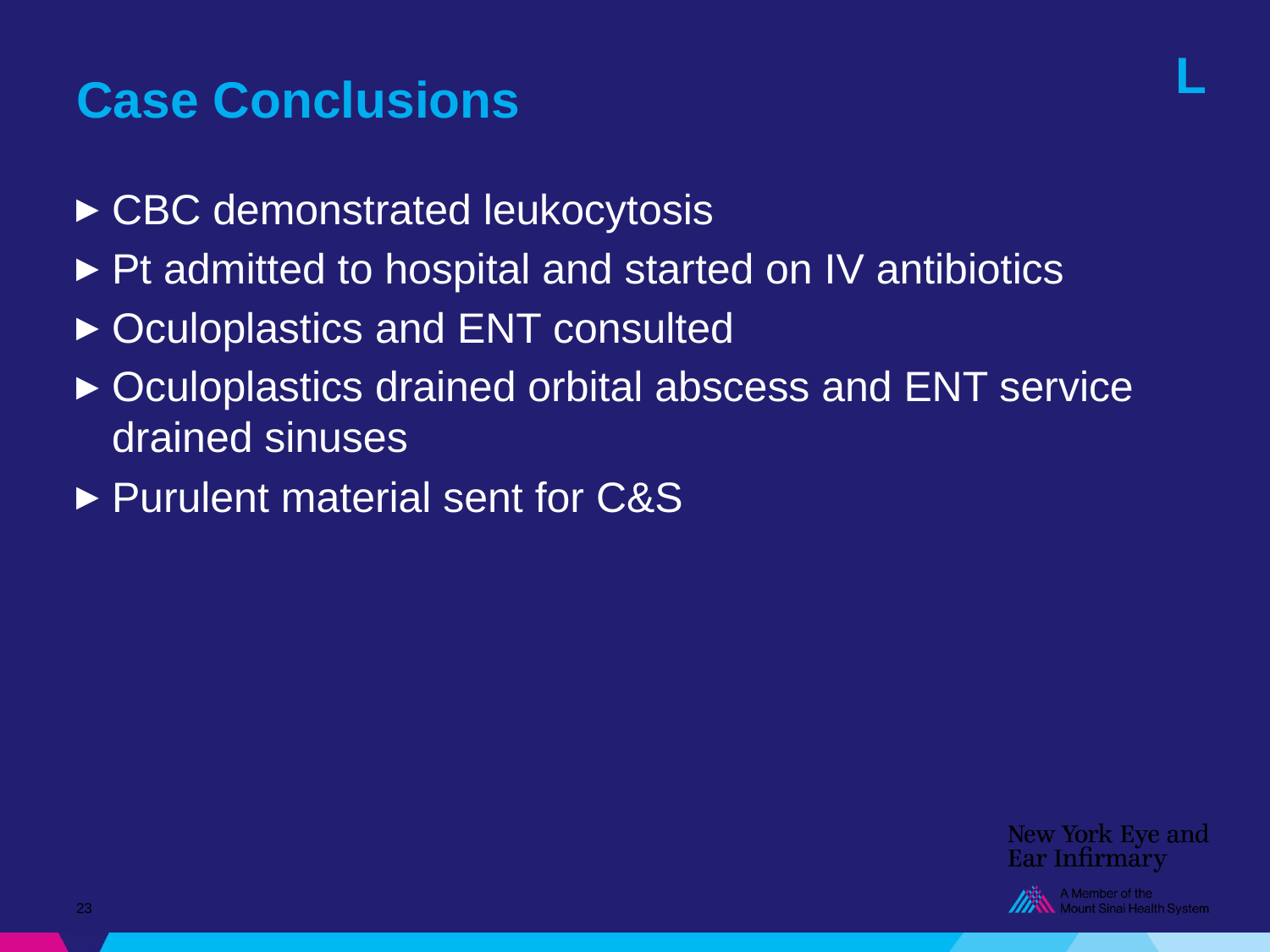

L
# Case Conclusions
CBC demonstrated leukocytosis
Pt admitted to hospital and started on IV antibiotics
Oculoplastics and ENT consulted
Oculoplastics drained orbital abscess and ENT service drained sinuses
Purulent material sent for C&S
23

## Slide 24
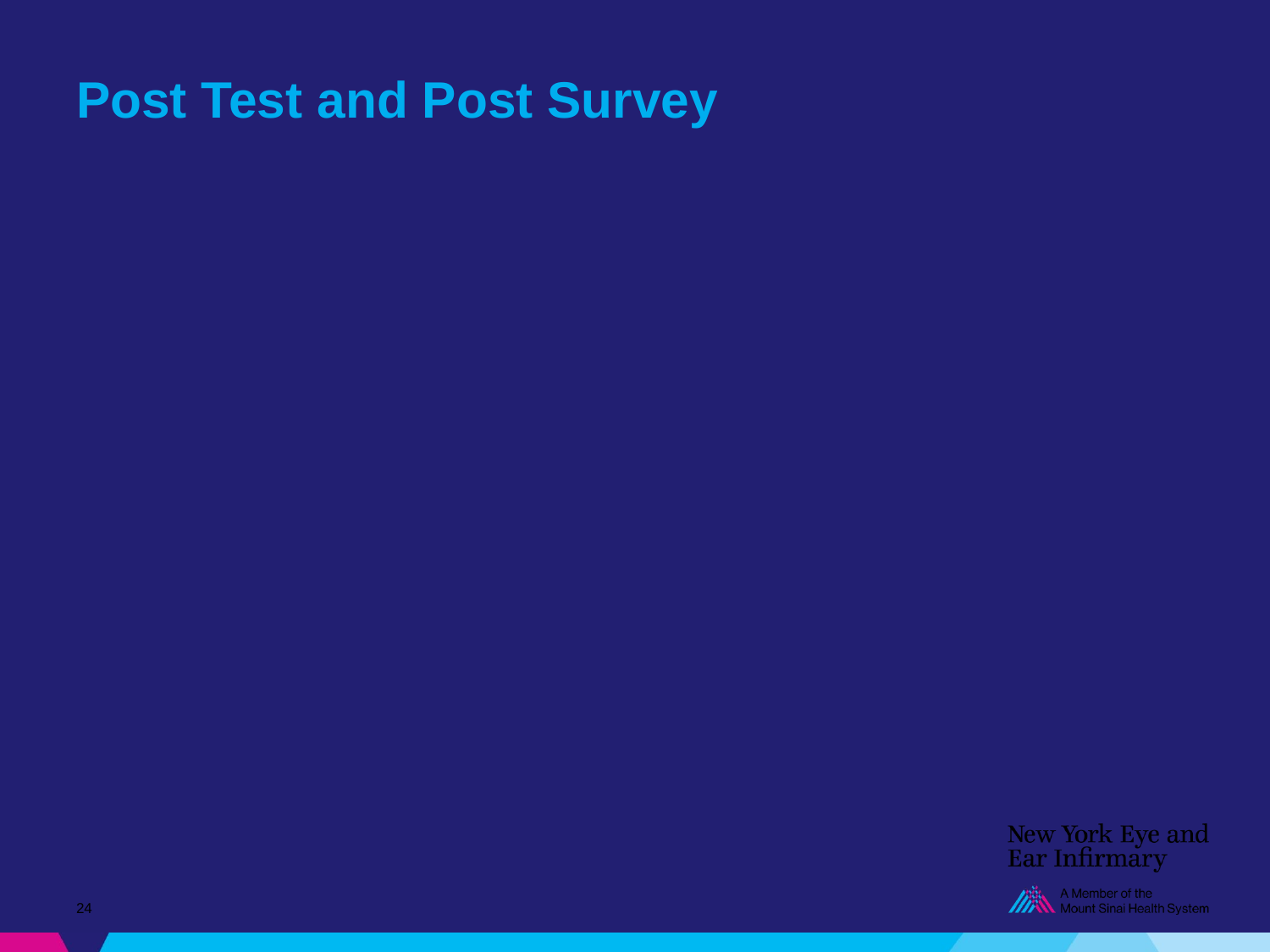

# Post Test and Post Survey
24
