# Supplementary material for: A Virtual Clinical Reasoning Case for Medical Students Using an Ophthalmology Model: A Case of Red Eye
Source: MedEdPORTAL. 2021 Mar 4;17:11117. doi: 10.15766/mep_2374-8265.11117 (PMC7970637; doi:10.15766/mep_2374-8265.11117)
Supplement: Supplementary file 1 — Faculty Guide.docxPre- and Posttest.docxTemplate for Google Document.docxRed Eye Clinical Reasoning Presentation.pptxRed Eye Session Polls.docx [file mep_2374-8265.11117-s001.zip › E. Red Eye Session Polls.docx]

**Polls**

**Poll#1: What is the most likely diagnosis?**

1. Blepharitis
2. Chalazion
3. Preseptal cellulitis
4. Orbital cellulitis
5. Dacryocystitis

**Poll #2 (optional post survey – 5 point Likert scale questions)**

1. How would you rate this session? (1-poor, 5 excellent)
2. How satisfied were you with the virtual learning design? (1-very unsatisfied, 5-very satisfied)
3. Would you recommend this session be repeated next year? (1- strongly do not recommend, 5- highly recommend)
